# Smart molecular design of NIR‐II organic fluorophores through self‐driven iterative evolution, deep learning, and fragment‐based assembly

**DOI:** 10.1002/smo2.70064

**Published:** 2026-06-10

**Authors:** Yu Zhang, Zhubin Hu, Xinyuan Wang, Zhenrong Sun, Yongye Liang, Cheng Zhong, Haitao Sun

**Affiliations:** ^1^ State Key Laboratory of Precision Spectroscopy School of Physics East China Normal University Shanghai China; ^2^ Department of Materials Science and Engineering Shenzhen Key Laboratory of Printed Organic Electronic Southern University of Science and Technology Shenzhen China; ^3^ Department of Chemistry Wuhan University Wuhan Hubei China; ^4^ Collaborative Innovation Center of Extreme Optics Shanxi University Taiyuan Shanxi China

**Keywords:** deep learning, fragment‐based assembly, molecular evolution, NIR‐II, TDDFT

## Abstract

Near‐infrared II (NIR‐II, 1000–1700 nm) emissive molecules are highly valued for biomedical imaging, phototherapy, and optoelectronic applications due to their deep tissue penetration, reduced autofluorescence, and high signal‐to‐noise ratio. However, their rational design remains challenging, as conventional discovery relies heavily on labor‐intensive synthesis, limited quantum chemical calculations, and inefficient trial‐and‐error exploration. To overcome these limitations, an iterative AI‐driven molecular evolution strategy (AI4NIR‐II 1.0) is introduced that integrates time‐dependent density functional theory (TDDFT), transformer‐based predictive modeling, and generative molecular design. Starting from donor‐acceptor‐donor (D‐A‐D) and donor‐donor‐acceptor‐donor‐donor (D‐D‐A‐D‐D) fragment scaffolds, a predictive model for both absorption and emission properties was trained based on a high quality dataset containing ∼16,000 molecules annotated by the optimally‐tuned range‐separated LC‐ωHPBE* (OTRS) functional. A fine‐tuned generative model was subsequently incorporated into a self‐refining‐loop workflow that cycles through molecular generation, property screening, and dataset augmentation. The model achieves excellent predictive performance for both emission and absorption properties, that is mean absolute errors of 19 nm for emission peak wavelengths and of 11 nm for absorption peak wavelengths, and correlation coefficient (*R* ^2^) exceeding 0.98 for wavelengths and oscillator strengths compared to OTRS‐TDDFT calculations. In addition, the resulting framework efficiently identifies promising NIR‐II candidates with accurate photophysical property predictions, and achieves speed improvements of three to four orders of magnitude over TDDFT calculations. Beyond accelerating NIR‐II fluorophore discovery, this self‐driven approach establishes a scalable and generalizable paradigm for NIR‐II molecular design, with applicability extending to optoelectronic materials and therapeutic compounds.

## Graphical abstract

A smart design strategy for NIR‐II organic fluorophores is proposed by combining self‐driven Iterative evolution, deep Learning, and fragment‐based assembly. This work establishes a broadly applicable approach for molecular design, accelerating the discovery of NIR‐II fluorophores and extending to optoelectronic materials and therapeutic compounds.

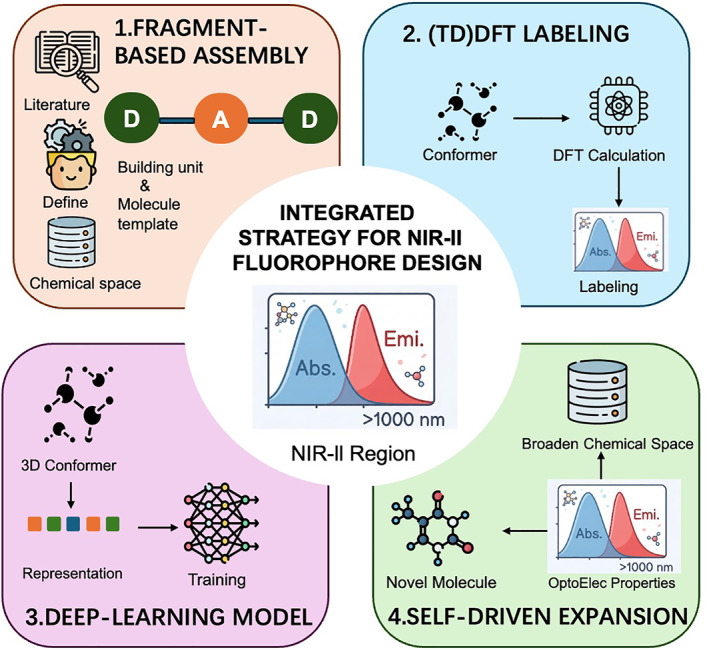

## 1 INTRODUCTION

Near‐infrared II (NIR‐II, 1000–1700 nm) emissive molecules have attracted considerable attention due to their wide‐ranging applications in biomedical imaging,[^1^, ^2^, ^3^] optoelectronic devices,^[4]^ phototherapy,^[5]^ etc. In particular, compared to the visible and the NIR‐I windows, their deeper tissue penetration, reduced autofluorescence, and higher signal‐to‐noise ratio make them indispensable for next‐generation fluorescence imaging and sensing platforms.[^6^, ^7^] However, the rational design of organic fluorophores efficiently operating in the NIR‐II window remains a challenge and the types of NIR‐II molecules are still limited. For instance, a class of high‐performance NIR‐II dyes rely on donor‐acceptor‐donor (D‐A‐D) or extended donor‐donor‐acceptor‐donor‐donor (D‐D‐A‐D‐D) architectures,[^8^, ^9^, ^10^] in which strong intramolecular charge transfer and acceptor engineering (e.g., benzo[1,2‐c:4,5‐c′]bis[1,2,5]thiadiazole (BBT), pyrrolo[3,4‐f]isoindole‐1,3,5,7(2H,6H)‐tetrone‐based quinoidal acceptors (PTQ), and thieno[3,2‐b]thiophene‐2,5‐dione‐based quinoidal acceptors (TQT)) are crucial for achieving long‐wavelength emission and stability.[^7^, ^11^, ^12^] However, designing such fluorophores with emission wavelengths in the NIR‐II window, accompanied by high quantum yield (QY), robustness, and biocompatibility, remains challenging, and conventional trial‐and‐error synthesis explores only a small fraction of the chemical space, which leaves a huge room for expansion.[^13^, ^14^, ^15^, ^16^]

Quantum chemical calculations, such as density functional theory (DFT) and time‐dependent DFT (TDDFT), have played an important role in guiding molecular design by providing predictive insights into photophysical properties. Although DFT exhibits favorable computational scaling for most medium‐sized organic molecules, NIR‐II fluorophores generally possess large π‐conjugated backbones, resulting in high computational cost for geometry optimization and particularly for excited‐state calculations based on thousands of large‐sized candidates.[^17^, ^18^] Therefore, even methods with relatively good scaling become impractical for large‐scale virtual screening of structurally diverse NIR‐II libraries.[^19^, ^20^] Consequently, only a limited portion of the chemical landscape can be explored by traditional quantum chemical calculations. This bottleneck has significantly hindered the pace of discovery, especially when both accuracy and diversity are required for practical applications.

Artificial intelligence (AI) techniques have recently opened new avenues for accelerating molecular discovery, offering the ability to bypass exhaustive quantum chemical calculations and experimental trial‐and‐error. Data‐driven predictive models have shown encouraging accuracy in estimating key photophysical properties, while generative algorithms are increasingly capable of proposing novel chemical structures beyond human intuition.[^21^, ^22^] However, despite these advances, most existing strategies face intrinsic limitations: predictive models alone cannot efficiently explore new chemical regions, and generative models without feedback often produce unrealistic or functionally irrelevant candidates. Moreover, these models are typically constrained by the quality and scope of their training datasets, which restricts their generalizability. Furthermore, many current approaches adopt a static one‐shot design paradigm, lacking iterative refinement and integration with domain knowledge. These challenges highlight the need for a self‐refining‐loop adaptive framework that unifies accurate prediction with guided molecular generation, enabling systematic exploration and progressive evolution of chemical space.[^20^, ^23^]

To address these limitations, herein, an integrated and modular workflow for data‐driven molecular discovery (AI4NIR‐II 1.0) is proposed, unifying fragment‐based chemical‐space construction, high‐fidelity quantum‐chemical labeling, deep‐learning property prediction, and adaptive molecular generation. Starting with curated fragment libraries equipped with explicit connection rules, the workflow programmatically assembles chemically interpretable structures, which are subsequently subjected to three‐dimensional (3D) conformer sampling and TDDFT‐based excited‐state characterization to produce high‐quality training data. These rigorously labeled structures serve as the foundation for predictive models capable of rapidly estimating key photophysical properties at scale. Guided by these predictions, an iterative expansion module identifies high‐value candidate molecules and seeds subsequent rounds of structure generation, thereby progressively increasing both structural diversity and functional relevance. Finally, to facilitate the use of potential users, we demonstrated a user‐friendly interface with AI4NIR‐II prediction capability.

## 2 COMPUTATIONAL DETAILS

### 2.1 Molecular library construction and data generation

**FIGURE 1:**
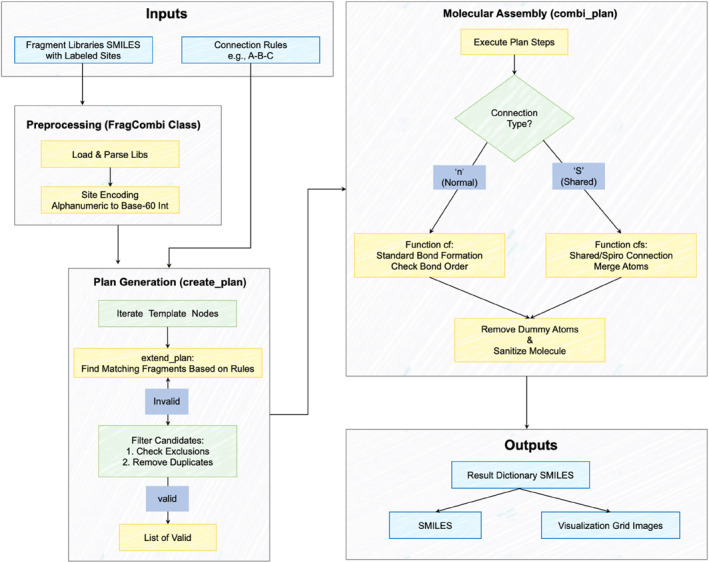
Structural logic and algorithmic workflow of the FragCombi framework.

The operational architecture of the home‐built FragCombi script follows a systematic hierarchical workflow, transitioning from raw fragment data to a refined virtual chemical space (Figure 1). This process begins with a Preprocessing Phase, where the system ingests fragment libraries formatted as simplified molecular input line entry system (SMILES) strings containing semantic site labels. To maintain the integrity of complex topological instructions within the RDKit^[24]^ environment, a specialized base‐60 encoding algorithm converts alphanumeric site identifiers into unique integer‐based atom map numbers. Upon entering the Plan Generation Phase, the algorithm executes an iterative search guided by a user‐defined structural template. The extend_plan function serves as the central logic engine, identifying compatible monomers through the validation of connection rules—specifically match_site for standard covalent bonding and shared_site for spiro or fused ring systems. During this stage, the framework concurrently applies a modification filter (modiflib) to resolve site exclusions and a duplication check (not_dup) to eliminate redundant topological paths, ensuring that only chemically viable assembly plans proceed. Finally, in the Molecular Assembly Phase, these validated plans are translated into molecular graphs. The script distinguishes between two primary ligation modes: the cf function for standard bond formation and the cfs function for atom‐sharing architectures. The process concludes with a sanitization step that removes auxiliary dummy atoms to produce standardized high‐quality SMILES outputs suitable for further computational analysis.

Thus, the FragCombi framework can be regarded as a general‐purpose molecular enumeration engine for the systematic construction of chemical space from modular building blocks. It enables automated fragment growth, fragment linking, and scaffold replacement under explicit connectivity, valence, and exclusion rules, ensuring chemically consistent graph assembly. This capability makes FragCombi well suited for optoelectronic molecular design; it can be employed to generate conjugated molecular architectures through the controlled assembly of π‐conjugated fragments, facilitating the exploration of structure–property relationships in organic semiconductors, emitters, and charge‐transport materials.

Herein, we focus on the D‐A‐D and D‐D‐A‐D‐D frameworks as the structural basis of the NIR‐II fluorophore data set, which provide a well‐defined and modular structural motif and allow systematic tuning of the electronic structure through independent modification of donor or acceptor strengths and π‐conjugation length. Such modularity makes D‐A‐D and D‐D‐A‐D‐D frameworks particularly suitable for data set construction and data‐driven molecular design, as they enable efficient exploration of chemically meaningful subspaces while avoiding the combinatorial explosion associated with unconstrained molecular enumeration. Thus, a diverse chemical library of D‐A‐D and D‐D‐A‐D‐D type molecules was constructed through the home‐built fragment‐based combinatorial algorithm (FragCombi script), assembling curated donor and acceptor fragments (shown in Supporting Information S1: Figures S1 and S2) based on predefined connection rules (see Supporting Information for details). The donor and acceptor fragments were collected from representative NIR‐II fluorophores reported in the literature and were further filtered to ensure synthetic accessibility and compatibility within conjugated backbones. The initial 3D geometries for all structures were generated through systematic conformational sampling using RDKit. Specifically, explicit hydrogen atoms were added, and up to 100 initial conformers per molecule were generated using the ETKDGv2 algorithm. A root‐mean‐square deviation (RMSD) threshold of 0.5 Å was applied to ensure structural diversity and eliminate redundant conformers. Each initial conformer was then subjected to geometry optimization for a maximum of 1000 iterations using the MMFF94 force field. The universal force field (UFF) was employed as an automatic fallback in cases where specific MMFF94 parameters were unavailable. The lowest‐energy conformers were selected as the optimal starting structures for subsequent ground‐state (S_0_) and excited‐state (S_1_) calculations. Absorption/emission peak wavelengths and corresponding oscillator strengths were subsequently calculated using TDDFT to create a labeled training set. Ground‐state (S_0_) and lowest excited‐state (S_1_) geometries were first optimized using B3LYP[^25^, ^26^]/6‐31G(d)^[27]^ method with Grimme's D3 (BJ) dispersion correction^[28]^. An optimally tuned range‐separated LC‐ωHPBE*/6‐31G(d) method^[29]^ was used to obtain emission wavelengths based on their S_1_‐optimized geometries. Notably, compared to conventional hybrid DFTs, the range separated hybrid functionals with an optimally tuned range separation parameter ω provides reliable descriptions for charge‐transfer‐dominated excitations, particularly in those D‐A‐D type organic dyes, as demonstrated by previous studies.[^30^, ^31^, ^32^, ^33^, ^34^] All calculations were performed using the Gaussian 16 program.^[35]^

### 2.2 Deep learning and iterative generative modeling

For property prediction, a deep learning model based on the Uni‐Mol[^36^, ^37^] was implemented as a regression task. Such a model was trained on a dataset comprising 15,940 molecules for emission wavelength prediction and 15,895 molecules for absorption wavelength prediction. The difference in the number of molecules stems from the convergence failure of some Gaussian computational tasks. A standard random 9:1 train‐test split was applied to the successfully processed data for model development and evaluation. The training set distribution for both emission and absorption peak wavelengths (Supporting Information S1: Figures S3 and S4) indicates that most molecules are within the range of 2000 nm. In theory, the model is expected to achieve predictive performance within this domain; however, predictions beyond this range should be regarded as extrapolations with epistemic uncertainty and treated as high‐risk candidates that need to be treated with care. The key hyperparameters are summarized in Supporting Information S1: Table S1.

To drive the discovery of novel NIR‐II emitters, a directed evolution strategy for chemical space was implemented. A conditional generative model (cMolGPT),^[38]^ pre‐trained on the SMILES strings, was fine‐tuned using molecules identified as NIR‐II emitters with their emission peak wavelengths ranging between 1000 and 2000 nm. This initiated an iterative loop: cMolGPT generated novel molecules, which were screened by the predictive model, and newly identified NIR‐II emitters were used to update the fine‐tuning set for the next round. The initial set of NIR‐II molecules was defined as pool0, with two successive expansion rounds yielding pool1 and pool2. The structural diversity and the expansion of chemical space across iterative generations of the molecular pools (pool0, pool1, pool2) were systematically evaluated using principal component analysis (PCA),^[39]^ clustering based on extended‐connectivity fingerprints (ECFPs),^[40]^ and Tanimoto similarity analysis.^[41]^ More computational details can be found in the Supporting Information.

## 3 RESULTS AND DISCUSSION

### 3.1 Overview of the molecular discovery and design workflow

**FIGURE 2:**
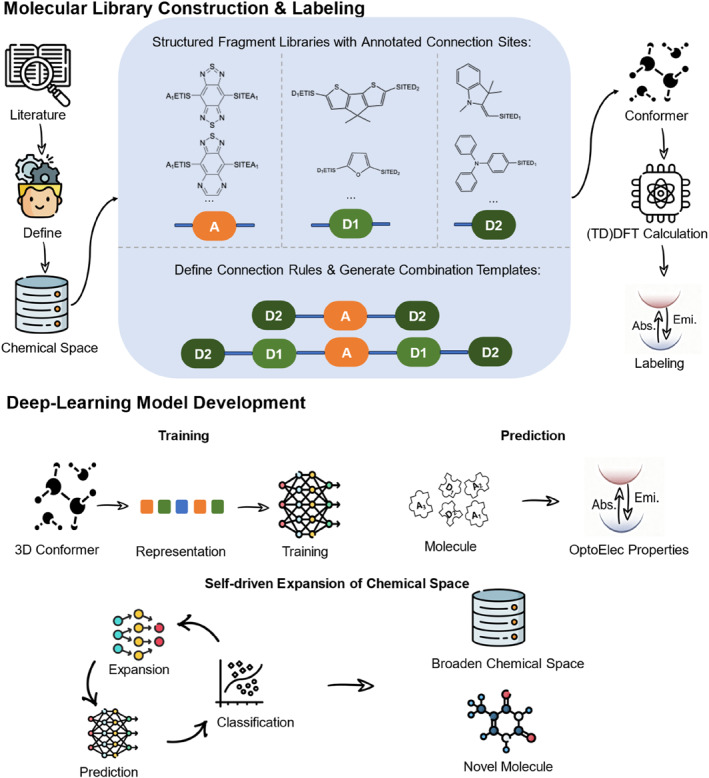
Schematic overview of the data‐generation and model‐application workflow.

As seen in Figure 2, the illustrated workflow presents an integrated modular strategy for data‐driven molecular discovery, combining fragment‐based chemical space construction, high‐quality quantum‐chemical labeling, deep‐learning model development, and self‐driven molecular generation. Firstly, the workflow begins with the systematic construction of a molecular library. Structured fragment libraries are curated from literature sources and enriched with annotated connection sites, enabling controlled assembly of donor‐acceptor‐donor (D‐A‐D) and related motifs. By defining explicit connection rules and generating permissible combination templates, the framework programmatically enumerates diverse molecular candidates, thereby expanding the accessible chemical space in a chemically interpretable manner. For each assembled structure, conformer sampling is performed to generate plausible 3D geometries, which are then subjected to DFT and TDDFT calculations. The resulting optimized structures and computed excited‐state properties—such as absorption and emission characteristics—are used to label the molecular library, providing high‐fidelity training data for downstream modeling.

Based on this labeled dataset, deep‐learning models are developed following a two‐stage pipeline. Molecular conformers are first converted into machine‐readable representations, which serve as inputs for model training. The trained models subsequently enable property prediction directly from molecular structures, thereby drastically reducing the computational cost compared to (TD)DFT‐based evaluations. This predictive capability provides a scalable means to screen large libraries and assess photophysical or optoelectronic properties with high throughput.

To further accelerate molecular discovery, a self‐driven chemical space expansion module was implemented. In this iterative loop, the trained model predicts properties for newly generated molecules, which are then classified based on task‐specific criteria. Promising candidates are fed back as seeds for subsequent molecular generation, enabling iterative refinement and exploration of the chemical space. Through this closed‐loop procedure by combining prediction, selection, and expansion, the framework autonomously drives the discovery of novel molecules while continuously broadening the underlying chemical space.

### 3.2 Predictive performance analysis

**FIGURE 3:**
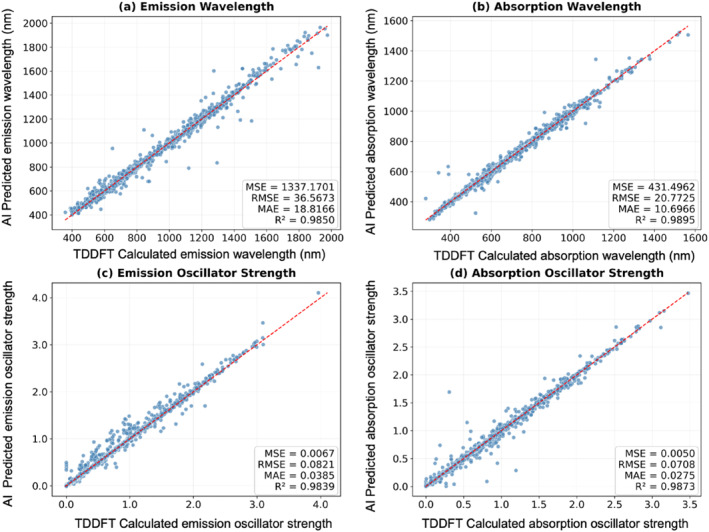
Predictive performance characterized by mean squared error, root mean square error, mean absolute error and correlation coefficient (*R* ^2^) for (a) emission peak wavelength and (c) oscillator strength, (b) absorption peak wavelength and (d) oscillator strength obtained by the AI‐model predicted values versus time‐dependent density functional theory calculated values.

As shown in Figure 3, a comparative analysis of TDDFT‐calculated and AI‐model predicted values for various molecular properties, that is, emission/absorption peak wavelengths and corresponding oscillator strengths, can be performed. Our previous studies demonstrate that the TDDFT calculation using the optimally tuned range‐separated hybrid functional method reliably describes charge‐transfer‐dominated excitations particularly evident in D‐A‐D type organic dyes, where it offers a notable improvement over conventional hybrid DFTs.[^30^, ^32^, ^34^] First, the near‐perfect alignment of data points with the ideal fit line confirms the model provides robust predictions for emission properties. The strong correlation (*R* ^2^ = 0.9850 and 0.9895) between TDDFT‐calculated and model‐predicted emission and absorption wavelengths indicates its robust predictive capability of the proposed model. The predicted mean squared errors (MSEs), root mean square errors (RMSEs), and mean absolute errors (MAEs) of emission/absorption peak wavelengths are 1337 nm^2^/431.50 nm^2^, 37 nm/21 nm, 19 nm/11 nm, respectively. Such errors are entirely acceptable, especially in the NIR region. Meanwhile, the high correlation coefficients (*R* ^2^ = 0.9839 and 0.9873) indicate that the model also performs well in predicting emission/absorption oscillator strengths. The analysis shows that this model not only reproduces theoretical values with high fidelity but also shows stable generalization across different property types.

In addition, the experimental values for selected NIR‐II molecules as reported in the literature are also listed in Table 1 to compare with the predicted values. The predictive absolute errors between the experimental and predictive values are ranged from 5 to 90 nm in wavelength units. These errors are converted into energy units in the NIR region ranging from only 0.01–0.11 eV, belonging to completely acceptable chemical precision. Overall, the results highlight that the model is reliable for practical molecular property prediction and possesses strong potential for application in molecular design, high‐throughput virtual screening, and the accelerated discovery of functional optoelectronic molecules.

**TABLE 1:** Comparison between emission peak wavelengths predicted by the model developed in this work and experimental values reported in the literature for selected NIR‐II molecules.

Selected molecules		λ_exp (nm)	λ_pred (nm)	Abs. error (nm)	Refs.
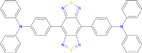	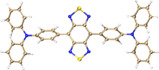	1055	965	90	[42]
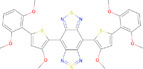	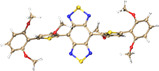	1047	1127	80	[43]
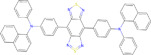	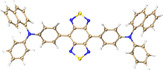	949	965	16	[44]
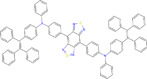	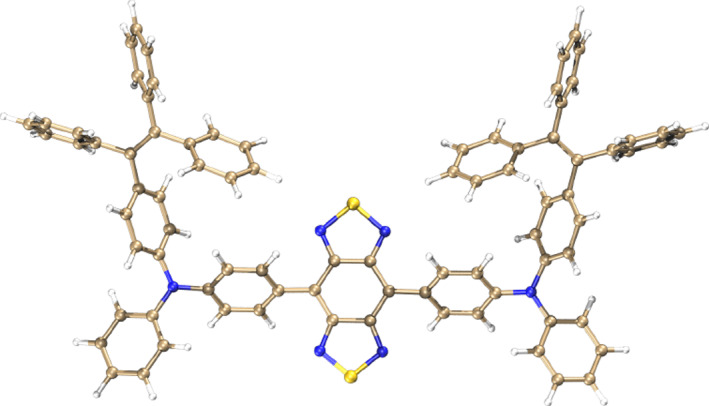	975	967	8	[45]
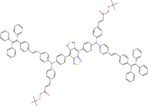	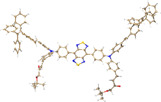	1000	1008	8	[46]
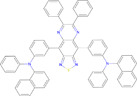	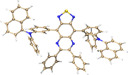	949	929	19	[47]
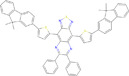	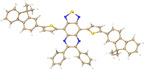	1073	1123	50	[48]
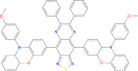	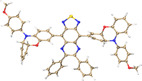	911	880	31	[49]
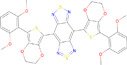	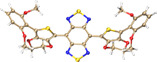	1025	1020	5	[50]
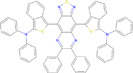	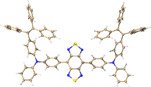	1125	1131	6	[51]

Besides the predictive accuracy of the proposed AI‐based model, the computational efficiency is also evaluated as a key parameter for the data‐driven models in molecular discovery. As summarized in Supporting Information S1: Table S2, a direct comparison of the computational cost between conventional TDDFT calculations and the proposed model reveals a dramatic improvement in efficiency. While TDDFT calculations require several days to more than 2 weeks of CPU time per molecule, such a model consistently produces predictions within tens of seconds to a few minutes. This results in speed‐up factors ranging from approximately 10^3^ to over 1.7 × 10^4^ corresponding to a reduction of computational time by more than three to four orders of magnitude. Note that such a substantial acceleration enables rapid, high‐throughput screening of molecular emission properties that would be computationally prohibitive using first‐principles methods alone.

### 3.3 Comparative analysis of chemical space coverage and diversity

**FIGURE 4:**
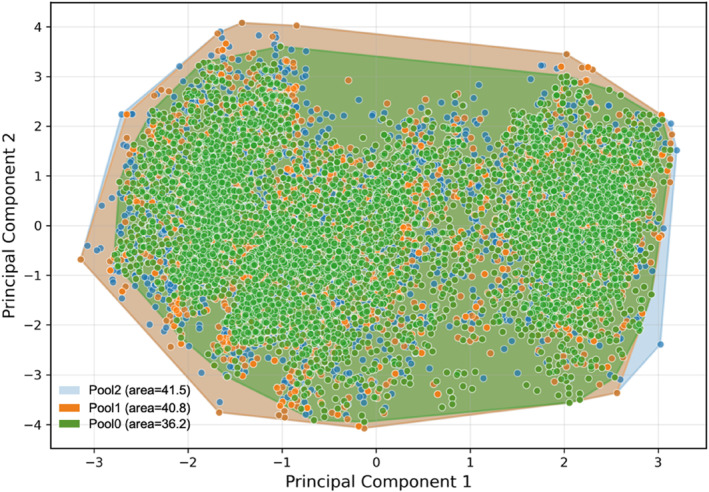
Expansion of chemical space coverage across iterative molecular pools. Convex hull areas computed from PCA‐projected molecular embeddings for three iterative molecular pools (pool0, pool1, and pool2). The progressive increase in the convex hull area—from pool0 to pool2—demonstrates the systematic expansion of the accessible chemical space. The inset shows the convex hull boundaries and molecular distributions for each pool in the reduced dimensionality space.

To quantitatively assess how iterative dataset construction expands chemical space, the coverage of each molecular pool (i.e., pool0, pool1, and pool2) using convex hull analysis in PCA‐reduced space was evaluated. As shown in Figure 4, the convex hull area increases monotonically from pool0 to pool2, reflecting a steady enlargement of the occupied chemical space. Pool0 consisting of 3158 clusters (similar molecular clustering) exhibits the smallest geometric footprint, consistent with its limited structural variety. Pool1 with 3864 clusters shows a pronounced increase in area, indicating that the first round of molecular augmentation effectively broadens exploration into new regions of the chemical landscape. Molecules in pool2 (4914 clusters) populate a wider and more heterogeneous region of the PCA space, enclosed by a noticeable 27% increase compared to pool0 and pool1, suggesting that the second augmentation cycle further diversifies the dataset and incorporates additional peripheral chemical structures that were absent in earlier pools.

In addition, some unique structures are captured in pool 2, which are not observed in pool1, demonstrating that pool2 is capable of sampling marginal regions of the chemical space that would otherwise remain unexplored. The result provides a geometric, model‐agnostic measure of chemical space growth and demonstrates that the iterative molecular generation strategy successfully enhances overall coverage and diversity in a cumulative manner.

### 3.4 Exploration of novel chemical space with promising candidates

**FIGURE 5:**
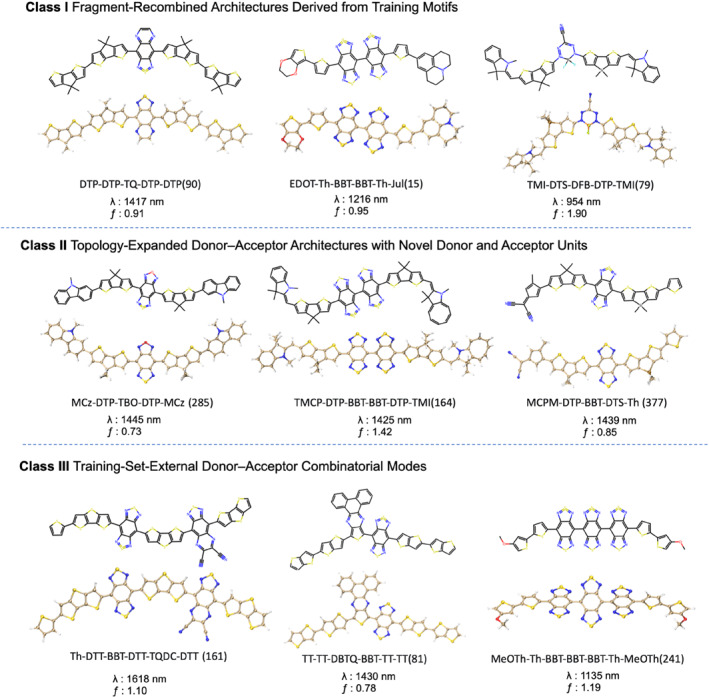
Representative AI‐generated NIR‐II fluorophores selected from the top‐ranked candidates according to the λ·ƒ metric defined in the maintext. The abbreviation (molecule ID in parentheses) and full names for the listed molecules are listed in Supporting Information S1: Table S3.

To comprehensively evaluate the generative performance of the fine‐tuned model, the top 60 generated molecules simply ranked by the product of emission peak wavelength (λ) and oscillator strength (ƒ) were examined as listed in Supporting Information S1: Figure S5. This composite metric simultaneously emphasizes long‐wavelength emission within the NIR‐II window and high radiative transition probability—both critical for high‐performance NIR‐II fluorophores. It can be seen that all those top‐ranked molecules are absent from the training data, confirming that they represent novel candidates rather than reproductions or near‐duplicates. The representative generated molecules were divided into three categories as shown in Figure 5.

Class I molecules are constructed through fragment recombination of molecular motifs derived from the training set, yet assembled in previously unobserved configurations. While the individual donor, acceptor, or π‐bridge fragments can be traced back to structural elements present in the training data, their spatial arrangement and connection pattern are distinct. For example, the resulting molecules preserve the symmetrical structural construction of D‐D‐A‐D‐D but possess new donor combinations. This indicates that the model is capable of learning fragment‐level representations and recombining them in chemically meaningful ways rather than reproducing intact molecular patterns. Such fragment‐level creativity is particularly valuable for molecular discovery, as it enables systematic exploration of nearby but previously inaccessible regions of chemical space while maintaining chemical plausibility and favorable photophysical performance.

Class II molecules represent topology‐expanded donor–acceptor architectures that retain a D‐D‐A‐D‐D‐like backbone while incorporating previously unseen donor and acceptor units. Although the overall donor–acceptor sequence resembles that of the training set, the individual donor and acceptor motifs differ substantially in their chemical identity and electronic characteristics. In particular, the model introduces newly combined heteroaromatic donors and electron‐deficient acceptors that were not explicitly present in the training data. These structures demonstrate that the model does not merely reproduce symmetrical D‐D‐A‐D‐D patterns but is capable of recombining donor and acceptor functionalities at a deeper chemical level. For example, the resulting molecules consist of asymmetrical π‐conjugation backbone such as D_1_‐D‐A‐D‐D_2_, maintaining strong intramolecular charge‐transfer character and extended conjugation, enabling efficient NIR‐II emission despite their increased structural novelty. This result indicates that the model has learned transferable electronic design principles rather than rigid structural templates.

Class III molecules exhibit donor–acceptor combinatorial modes that are entirely absent from the training dataset. Unlike the typical D‐D‐A‐D‐D or D‐A‐D arrangements, these structures feature alternative sequences and spatial organizations of donor and acceptor units such as D‐A‐D‐A‐D, D‐D‐A‐A‐D‐D, D‐D‐A‐A‐A‐D‐D. First, the emergence of such new architectures indicates that the model is able to transcend the explicit connectivity rules present in the training data and explore new combinatorial patterns guided by photophysical objectives. Notably, despite deviating from known donor–acceptor arrangements, a structural configuration consisting of an acceptor dimer and trimer is proposed, retaining pronounced NIR‐II emission and sizable oscillator strengths.

Overall, the fine‐tuned model exhibits a hierarchical and progressively advanced generative capability, as revealed by the three classes, which expands the NIR‐II chemical space across multiple structural hierarchies—from unit substitution to topology reorganization and fragment recombination—signifying principled molecular innovation rather than simple memorization. Class I highlights fragment‐level recombination, assembling learned motifs into previously unobserved yet chemically coherent structures. Class II demonstrates unit‐level diversification within familiar topologies. Class III shows architectural extrapolation through novel donor–acceptor connectivities absent from the training set.

Furthermore, to ensure the practical relevance of the AI‐driven molecular evolution, the retrosynthetic feasibility of the generated candidates was systematically evaluated utilizing the RDKit synthetic accessibility score (SAScore). Typically, the SAScore evaluates synthetic complexity on a scale from 1 (extremely easy to synthesize) to 10 (extremely difficult). As illustrated by the SAScore distribution of the generated pool (Supporting Information S1: Figure S6), the AI‐proposed NIR‐II candidates exhibit good chemical synthesizability. Notably, over 76.2% of the candidates feature highly favorable SAScores below 4.5, and an overwhelming 96.5% of the molecules score strictly below 5.0, a regime typically associated with straightforward multistep synthesis. Given the inherently extended π‐conjugated networks present in highly efficient NIR‐II D‐D‐A‐D‐D fluorophores, this distribution clearly indicates that the model successfully avoids generating chemically unreasonable structures, highlighting the robustness of the FragCombi fragment‐based algorithmic design.

### 3.5 Accessible online interface for AI4NIR‐II 1.0

Finally, to facilitate direct use of the AI4NIR‐II 1.0 functionalities by potential users, we have also implemented its AI prediction capabilities as an accessible online interface (http://www.ai4optoelectro.com/). This platform enables rapid prediction of absorption and emission wavelengths and oscillator strengths, and provides functionality for querying both the training dataset and the model‐generated molecular dataset using wavelength and oscillator strength as search criteria. For details, please refer to the video demonstration in Video S1.

## 4 CONCLUSION

In this work, a self‐refining‐loop AI‐driven strategy is developed for the autonomous discovery of NIR‐II emissive molecules. By integrating DFT‐based data generation, transformer‐based property prediction, and iterative generative modeling, the framework systematically explores chemical space and efficiently identifies candidates with desirable photophysical properties. The iterative design paradigm enables continuous expansion and refinement of the molecular library, resulting in both structural diversity and functional relevance. Compared with traditional synthesis‐driven or DFT approaches, this methodology provides substantial advantages in scalability, efficiency, and adaptability. This unified workflow leverages fragment‐based chemical design, quantum‐chemical labeling, and adaptive AI‐guided exploration to efficiently identify structurally diverse molecular candidates with desirable properties.

It should be noted, however, that the current implementation focuses primarily on the prediction of emission wavelength and oscillator strength, while other critical photophysical parameters, such as brightness, QY, complex radiative and non‐radiative transition processes, and vibronic coupling effects, are not yet accounted for. These properties are essential for comprehensive performance evaluation and practical application, and will be incorporated in the forthcoming version 2.0 of the current framework. Additionally, future model refinement could viably incorporate auxiliary descriptors such as solvent‐related parameters, 3D conformational features, and electronic structure descriptors. Furthermore, the proposed concept of iterative molecular evolution herein is not limited to NIR‐II fluorophores and can be generalized to a broad range of functional molecules extended to other optoelectronic materials and drug‐like chemical spaces.

## CONFLICT OF INTEREST STATEMENT

The authors declare no conflicts of interest.

## ETHICS STATEMENT

No animal or human experiments were involved in this study.

## Supporting information

Supporting Information S1

Supporting Information S2

Video S1

## Data Availability

All data supporting the findings of this study are available within the article as well as the Supplementary Information file or available from the corresponding authors on reasonable request.

## References

[1] M. Zhao , X. Chen , Acc. Mater. Res. 2024, 5, 600.

[2] X. Zhang , Z. Xi , D. Zhang , et al., Chin. Chem. Lett. 2026, 37, 111041.

[3] G. Hong , A. L. Antaris , H. Dai , Nat. Biomed. Eng. 2017, 1, 0010.

[4] Y. Yu , X.‐Y. Xia , C.‐F. Xu , et al., J. Am. Chem. Soc. 2024, 146, 11845. 38648548 10.1021/jacs.4c00648

[5] H. Zhou , X. Zeng , A. Li , et al., Nat. Commun. 2020, 11, 6183. 33273452 10.1038/s41467-020-19945-w PMC7713230

[6] Y. Liu , Y. Li , S. Koo , et al., Chem. Rev. 2022, 122, 209. 34664951 10.1021/acs.chemrev.1c00553

[7] F. Wang , Y. Zhong , O. Bruns , et al., Nat. Photonics 2024, 18, 535.

[8] W. Shao , Q. Wei , S. Wang , et al., Mater. Horiz. 2020, 7, 1379.

[9] J. Gao , L. Yuan , Y. Min , et al., Biomater. Sci. 2024, 12, 1320. 38273769 10.1039/d3bm01604h

[10] Q. Yang , H. Ma , Y. Liang , et al., Acc. Mater. Res. 2021, 2, 170.

[11] R. Tian , X. Feng , L. Wei , et al., Nat. Commun. 2022, 13, 2853. 35606352 10.1038/s41467-022-30304-9 PMC9127093

[12] N. E. Sparks , C. Smith , T. Stahl , et al., J. Mater. Chem. C 2024, 12, 4369.

[13] W. Wu , Y. Yang , Y. Yang , et al., Small 2019, 15, 1805549.

[14] Z. Feng , Y. Li , S. Chen , et al., Nat. Commun. 2023, 14, 5017. 37596326 10.1038/s41467-023-40728-6 PMC10439134

[15] L. Wang , N. Li , W. Wang , et al., ACS Nano 2024, 18, 4683. 38295152 10.1021/acsnano.3c12316

[16] S. He , J. Song , J. Qu , et al., Chem. Soc. Rev. 2018, 47, 4258. 29725670 10.1039/c8cs00234g

[17] S. Havenridge , R. Rüger , C. M. Aikens , J. Chem. Phys. 2023, 158, 224103. 37290069 10.1063/5.0142240

[18] C. A. Ullrich , APL Comput. Phys. 2025, 1, 020901.

[19] N. N. T. Pham , S. H. Han , J. S. Park , et al., Nanomaterials 2021, 11, 2293.34578610

[20] R. P. Joshi , N. Kumar , Molecules 2021, 26, 6761.34833853

[21] B. A. Koscher , R. B. Canty , M. A. McDonald , et al., Science 2023, 382, eadi1407. 38127734 10.1126/science.adi1407

[22] R. Li , Q. Ou , Z. Shuai , Chem. Soc. Rev. 2025, 54, 11699. 41247239 10.1039/d5cs00959f

[23] T. Khater , S. A. Alkhatib , A. AlShehhi , et al., J. Cheminformatics 2025, 17, 116. 10.1186/s13321-025-01059-4 PMC12323263 40759950

[24] G. Landrum , RDKit: Open‐Source Cheminformatics 2006.

[25] C. Lee , W. Yang , R. G. Parr , Phys. Rev. B 1988, 37, 785. 10.1103/physrevb.37.785 9944570

[26] A. D. Becke , Phys. Rev. A. 1988, 38, 3098. 10.1103/physreva.38.3098 9900728

[27] A. D. McLean , G. S. Chandler , J. Chem. Phys. 1980, 72, 5639.

[28] S. Grimme , S. Ehrlich , L. Goerigk , J. Comput. Chem. 2011, 32, 1456. 21370243 10.1002/jcc.21759

[29] T. M. Henderson , A. F. Izmaylov , G. Scalmani , et al., J. Chem. Phys. 2009, 131, 044108. 19655838 10.1063/1.3185673

[30] H. Sun , J. Autschbach , J. Chem. Theor. Comput. 2014, 10, 1035. 10.1021/ct4009975 26580181

[31] D. Jacquemin , B. Moore , A. Planchat , C. Adamo , J. Autschbach , J. Chem. Theor. Comput. 2014, 10, 1677. 10.1021/ct5000617 26580376

[32] Q. Yang , Z. Hu , S. Zhu , et al., J. Am. Chem. Soc. 2018, 140, 1715. 29337545 10.1021/jacs.7b10334

[33] C. Ju , E. J. French , N. Geva , et al., J. Phys. Chem. Lett. 2021, 12, 9516. 34559964 10.1021/acs.jpclett.1c02506

[34] H. Sun , C. Zhong , J.‐L. Brédas , J. Chem. Theor. Comput. 2015, 11, 3851. 10.1021/acs.jctc.5b00431 26574466

[35] M. J. Frisch , et al., Gaussian 16, Gaussian, Inc., Wallingford CT 2016.

[36] S. Lu , Z. Gao , D. He , et al., Nat. Commun. 2024, 15, 7104. 39160169 10.1038/s41467-024-51321-w PMC11333583

[37] G. Zhou , Z. Gao , Q. Ding , et al., in The Eleventh International Conference on Learning Representations 2023.

[38] Y. Wang , H. Zhao , S. Sciabola , et al., Molecules 2023, 28, 4430. 37298906 10.3390/molecules28114430 PMC10254772

[39] I. T. Jolliffe , Principal Component Analysis, Springer.

[40] D. Rogers , M. Hahn , J. Chem. Inf. Model. 2010, 50, 742. 20426451 10.1021/ci100050t

[41] D. Bajusz , A. Rácz , K. Héberger , J. Cheminformatics 2015, 7, 20. 10.1186/s13321-015-0069-3 PMC4456712 26052348

[42] A. L. Antaris , H. Chen , K. Cheng , et al., Nat. Mater. 2016, 15, 235. 26595119 10.1038/nmat4476

[43] H. Wan , H. Ma , S. Zhu , F. Wang , Y. Tian , R. Ma , Q. Yang , T. Zhu , W. Wang , Z. Ma , M. Zhang , Y. Zhong , H. Sun , Y. Liang , H. Dai , Adv. Funct. Mater. 2018, 28, 1804956. 31832053 10.1002/adfm.201804956 PMC6907024

[44] N. Alifu , A. Zebibula , J. Qi , et al., ACS Nano 2018, 12, 11282. 30345739 10.1021/acsnano.8b05937

[45] Z. Sheng , B. Guo , D. Hu , et al., Adv. Mater. 2018, 30, 1800766.

[46] P. Xu , F. Kang , W. Yang , et al., Nanoscale 2020, 12, 5084. 32068224 10.1039/c9nr09999a

[47] J. Qi , C. Sun , A. Zebibula , et al., Adv. Mater. 2018, 30, 1706856. 10.1002/adma.201706856 29341330

[48] K. He , S. Chen , Y. Chen , et al., ACS Appl. Polym. Mater. 2021, 3, 3238.

[49] S. Li , T. Cheng , C. Yin , et al., ACS Appl. Mater. Interfaces 2020, 12, 43466. 32907323 10.1021/acsami.0c12773

[50] R. Tian , H. Ma , Q. Yang , et al., Chem. Sci. 2019, 10, 326. 30713641 10.1039/c8sc03751e PMC6333232

[51] D. Yan , W. Xie , J. Zhang , et al., Angew. Chem. Int. Ed. 2021, 60, 26769. 10.1002/anie.202111767 34626441

