# Supplementary material for: Smart molecular design of NIR‐II organic fluorophores through self‐driven iterative evolution, deep learning, and fragment‐based assembly
Source: Smart Mol. 2026 Jun 10:e70064. Online ahead of print. doi: 10.1002/smo2.70064 (PMC13398758; doi:10.1002/smo2.70064)
Supplement: Supplementary file 1 — Supporting Information S1 [file SMO2-9999-0-s003.docx]

***Supporting Information for***

**Smart Molecular Design of NIR-II Organic Fluorophores via Self-Driven Iterative Evolution, Deep Learning, and Fragment-Based Assembly**

Yu Zhang^1^, Zhubin Hu^1^, Xinyuan Wang^2^, Zhenrong Sun^1^, Yongye Liang^2*^, Cheng Zhong^3*^ and Haitao Sun^1,4*^

^1^ *State Key Laboratory of Precision Spectroscopy, School of Physics,*

*East China Normal University, Shanghai 200241, China*

^2^ *Department of Materials Science and Engineering, Shenzhen Key Laboratory of Printed Organic Electronic, Southern University of Science and Technology, Shenzhen 518055, China*

^3^ *Department of Chemistry, Wuhan University, Hubei 430072, China*

^4^ *Collaborative Innovation Center of Extreme Optics, Shanxi University,*

*Taiyuan, Shanxi 030006, China*

*Corresponding author: htsun@phy.ecnu.edu.cn (H.S.); liangyy@sustech.edu.cn(Y.L.); zhongcheng@whu.edu.cn(C.Z.).

**Computational details**

To construct the chemical library of D-A-D and D-D-A-D-D structures, a molecular fragment combination algorithm was employed. This script implements an automated computational framework for fragment-based combinatorial chemistry, enabling systematic generation of novel chemical structures through algorithmic combination of molecular fragments. The core FragCombi class orchestrates a multi-step workflow: 1) Fragment libraries are loaded from structured text files with site-specific annotations; 2) Combinatorial plans are generated using a template-directed approach ("A-D1-D1" sequence) with rigorous connection rules governing site matching ([('A1','D1'), ('D2','D3'), ('A1','D3')]) and duplicate site handling (["A1","D2"]); 3) Molecular assembly executes through specialized connection functions that form validated chemical bonds while preserving valence constraints; 4) Generated structures undergo duplicate filtering and are output as both machine-readable JSON data and visualized molecular grids. The implementation leverages RDKit for chemical operations, employs base-60 site encoding for robust pattern matching, and incorporates combinatorial optimization techniques for efficient exploration of chemical space. This framework transformed fragment libraries into diverse, synthetically plausible compounds while maintaining chemical integrity through automated bond-order validation and site compatibility checks. The acceptors and donors used were shown in Figure S1and Figure S2.

Computational Methodology for Initial Conformer Generation: The conformational sampling and geometry optimization workflow was implemented in Python using RDKit (version 2023.03.1). For each compound, the SMILES string was first converted to a molecular structure with explicit hydrogens added. For each compound, 100 conformers were systematically generated to ensure comprehensive coverage of the potential energy surface. Structural diversity was maintained through application of a root-mean-square (RMS) pruning threshold of 0.5 Å during conformer generation. Molecular symmetry was rigorously preserved throughout the embedding process via RDKit's internal symmetry perception algorithms. Energy minimization employed a hierarchical force-field strategy with the Merck Molecular Force Field (MMFF94) serving as the primary optimization method using default parameters. When MMFF proved inapplicable (notably for molecules containing exotic elements), the Universal Force Field (UFF) was automatically substituted. All minimizations were conducted with a convergence criterion of gradient tolerance <0.001 kcal/mol·Å and terminated after a maximum of 2,000 iterations per conformer. Convergence: Maximum of 2,000 iterations per conformer (termination upon gradient tolerance <0.001 kcal/mol·Å). For post-optimization, conformational energies were evaluated using the respective force field's energy function. The lowest-energy conformer was selected and exported in XYZ format with atomic coordinates truncated to 4 decimal places. This dual force-field strategy ensured robust handling of diverse chemical space while maintaining computational efficiency through parallelization (multithreaded implementation).

Quantum Chemical Calculations: The ground-state (S_0_) geometries of all molecules were optimized using B3LYP 6-31G(d) method with Grimme's D3 (BJ) dispersion correction. The corresponding range separation parameter (ω, in Bohr^-1^) for each molecule was obtained by optimally tuned method. The excited-state (S_1_) geometries of these molecules were optimized using time dependent (TD)-tuned-LC-ωHPBE*/6-31G(d) method. The emission excitation energies of these molecules were calculated at the TD-tuned-LC-ωHPBE*/6-31G(d) level based on their optimized S_1_ geometries. All the calculations were performed using the Gaussian 16 program.

For property prediction, a deep learning model based on a transformer neural network architecture was implemented using the Uni-Mol framework. The model was configured as a regression task to predict a single target property, and automated mixed-precision (AMP) was employed to improve computational efficiency. For emission wavelength prediction, a total of 15,940 molecules were used, split into training and test sets with a 9:1 ratio. For absorption wavelength prediction, 15,895 molecules were similarly divided using a 9:1 train-test split. Key hyperparameters and training configurations are summarized in Table S1. The distribution of emission wavelengths for the 15,940 molecules in the training set is shown in Figure S3. The wavelengths span from 306 nm to well within the NIR-II region, with a significant portion (26%) of molecules emitting beyond 1000 nm. This direct representation of NIR-II emitters in the training data provides a foundational basis for the model's predictive reliability in this wavelength range. The wavelengths span of absorption wavelength is also shown in Figure S4.

Directed Evolution of Chemical Space: The emission wavelengths and oscillator strengths of 15,940 molecules were calculated. Based on this dataset, a deep learning model was trained using Uni-Mol to predict emission wavelengths and oscillator strengths. From these 15,940 molecules, those exhibiting emission wavelengths within the range of 1000-2000 nm was selected. Built upon a Transformer architecture, the cMOLGPT model is pre-trained extensively on SMILES representations of drug-like molecules and fine-tuned with ⟨compound, target⟩ pairs to achieve target-oriented generation. In this work, we employ this framework and fine-tune the model using molecules exhibiting predicted emission wavelengths within the NIR-II window as the training set, thereby enabling the model to generate molecules possessing NIR-II emission capabilities. Subsequently, cMOLGPT was employed to generate 6,000 novel molecules. These generated molecules were then screened based on predictions from the aforementioned predictive model to identify those with NIR-II emission characteristics. The newly identified NIR-II molecules were incorporated into the fine-tuning dataset. This process—generating 6,000 new molecules, predicting their properties, screening for NIR-II emitters, and updating the fine-tuning set—was iterated in a cyclic manner. The initial set of molecules with emission wavelengths greater than 1000 nm was defined as pool0, and the sets obtained after two successive rounds of generative expansion were designated as pool1 and pool2, respectively.

To systematically evaluate the coverage and diversity of the molecular sets (pool0, pool1, and pool2), a combination of dimensionality reduction, clustering, similarity, and coverage analyses was employed. These complementary approaches enabled both qualitative visualization and quantitative assessment of the extent to which each set explores distinct regions of the chemical space.

Chemical space projection was conducted using Principal Component Analysis (PCA) based on molecular descriptor vectors. PCA was applied to the full descriptor matrix, and the first two principal components were retained to capture the dominant variance and provide a global representation of the distribution of pool0, pool1, and pool2 in orthogonal chemical dimensions.

Chemical diversity was evaluated through clustering analysis derived from pairwise molecular structural similarity. Similarity matrices were computed using Extended-connectivity fingerprints (ECFPs) , and molecules were grouped into clusters such that structurally similar compounds were assigned to the same class. The number of clusters obtained for each dataset was used as a quantitative indicator of chemical diversity. In addition, the average number of molecules per cluster (cluster density) was calculated to assess the sampling density within each structural region.

Coverage analysis was performed by identifying clusters or molecules that were present in one dataset but absent in another. The relative proportion of such unique elements, normalized to the size of a reference pool, was used to quantify the incremental chemical space coverage contributed by each dataset.

Molecular similarity analysis was further conducted to assess both inter-set and intra-set structural relationships. The Tanimoto coefficient was employed to measure the similarity between two fingerprints A and B, defined as

$$\text{}$$

where a and b denote the number of bits set in A and B, respectively, and ccc denotes the number of shared bits. Inter-set similarity was calculated as the mean pairwise Tanimoto value between molecules from different pools, while intra-set similarity was computed as the average similarity among molecules within each pool. These metrics enabled assessment of structural redundancy and the degree to which additional pools introduce novel chemotypes.

Together, the PCA projection, clustering-based diversity analysis, coverage evaluation, and similarity assessment provided a systematic characterization of the chemical space occupied by pool0, pool1, and pool2, allowing quantitative comparison of their extent, diversity, and complementarity.

Table S1: Hyperparameters and Training Settings for the Uni-Mol Transformer Model

| Parameter | Value | Description |
| --- | --- | --- |
| Model Architecture |  |  |
| model_name | unimolv2 | Specific Uni-Mol model variant |
| model_size | 310m | Parameter size of the model |
| data_type | molecule | Input data type |
| remove_hs | false | Explicit hydrogens retained in representation |
| smi_strict | true | Strict SMILES parsing enforced |
| Task & Output |  |  |
| task | regression | Model task type |
| num_classes | 1 | Number of output nodes (single target regression) |
| target_cols | TARGET | Column name prefix for target property |
| target_normalize | auto | Automatic normalization applied to target values |
| Training Procedure |  |  |
| learning_rate | 1.0e-05 | Initial learning rate |
| batch_size | 8 | Samples per batch |
| epochs / max_epochs | 300 / 100 | Maximum training epochs (stopping condition likely via patience) |
| warmup_ratio | 0.03 | Fraction of steps for learning rate warmup |
| max_norm | 0.5 | Gradient clipping threshold |
| use_amp / amp | true | Automated Mixed Precision (AMP) enabled |
| cuda / use_cuda | true | GPU acceleration enabled |
| metrics | mse | Primary evaluation metric (Mean Squared Error) |
| patience | 400 | Early stopping patience (epochs without improvement) |
| Data Handling |  |  |
| split_method | 3fold_random | 3-fold cross-validation with random splitting |
| split_seed | 42 | Random seed for data splitting reproducibility |
| split_group_col | scaffold | Column used for scaffold-based splitting (context-dependent) |
| smiles_col | SMILES | Column name containing SMILES strings |
| kfold | 3 | Number of folds for cross-validation |
| Reproducibility |  |  |
| seed | 42 | Global random seed for reproducibility |
| anomaly_clean | false | Disabled PyTorch anomaly detection (typically for debugging) |
| freeze_layers | null | No layers frozen during training |
| freeze_layers_reversed | false | Layer freezing direction not applicable |
| load_model_dir | null | Training started from scratch (no pre-loaded model) |
| Logging |  |  |
| logger_level | 1 | Verbosity level for logging output |

|  |  |  |
| --- | --- | --- |
|  |  |  |
|  |  |  |
|  |  |  |
|  |  |  |
|  |  |  |
|  |  |  |
|  |  |  |
|  |  |  |
|  |  |  |
|  |  |  |
|  |  |  |
|  |  |  |
|  |  |  |

Figure S1. List of acceptors used in molecular space generation.

|  |  |  |  |
| --- | --- | --- | --- |
|  |  |  |  |
|  |  |  |  |
|  |  |  |  |
|  |  |  |  |
|  |  |  |  |
|  |  |  |  |
|  |  |  |  |
|  |  |  |  |
|  |  |  |  |
|  |  |  |  |
|  |  |  |  |
|  |  |  |  |
|  |  |  |  |
|  |  |  |  |
|  |  |  |  |

Figure S2. List of donors used in molecular space generation.


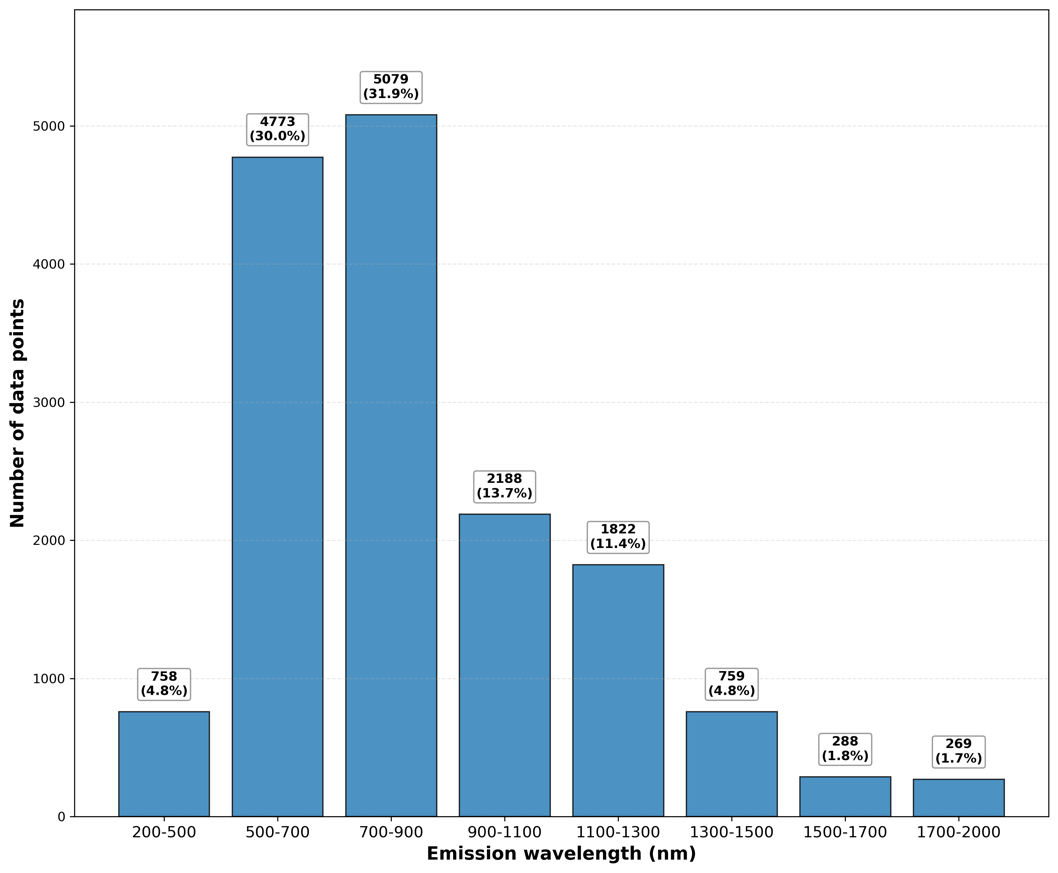


Figure S3. The distribution of emission wavelengths.


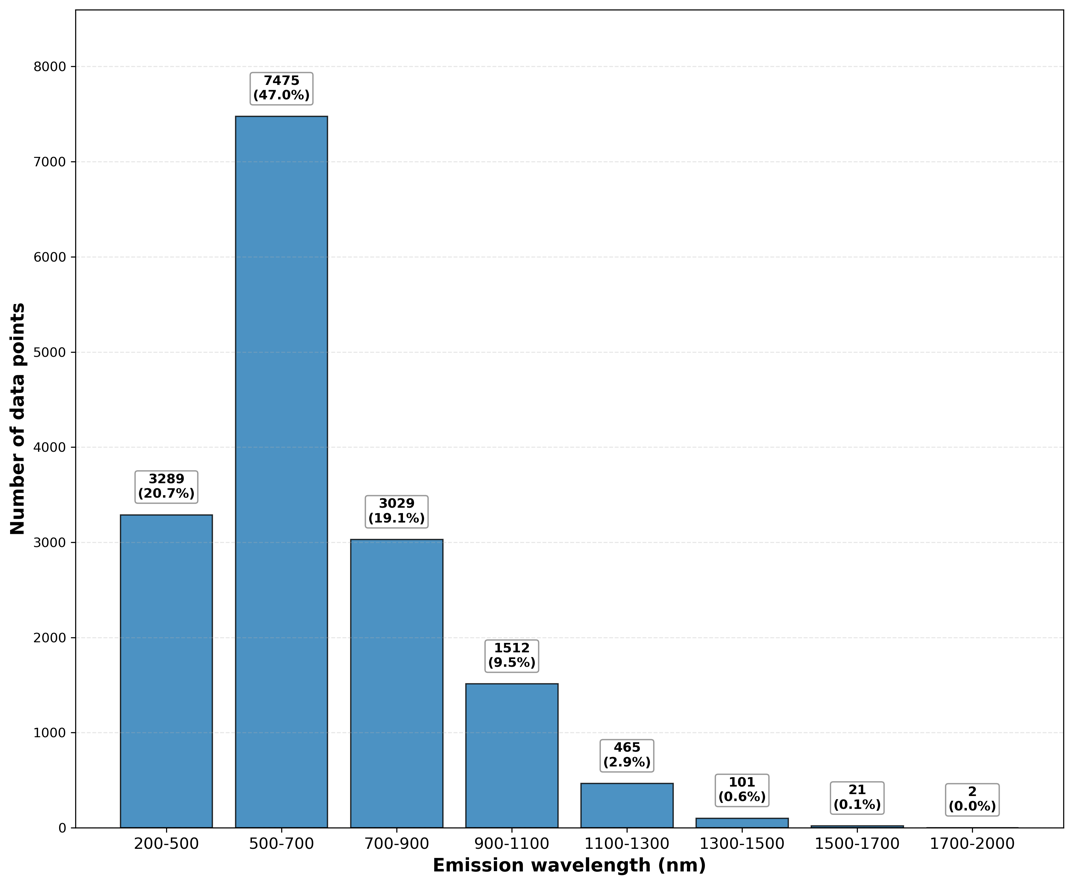


Figure S4. The distribution of absorption wavelengths.

Table S2. Comparison of computational cost between TDDFT calculations and the model predictions for molecular emission wavelengths.

| Selected Molecules | TDDFT CPU Time | Model Inference Time | Speed-up (×) |
| --- | --- | --- | --- |
| 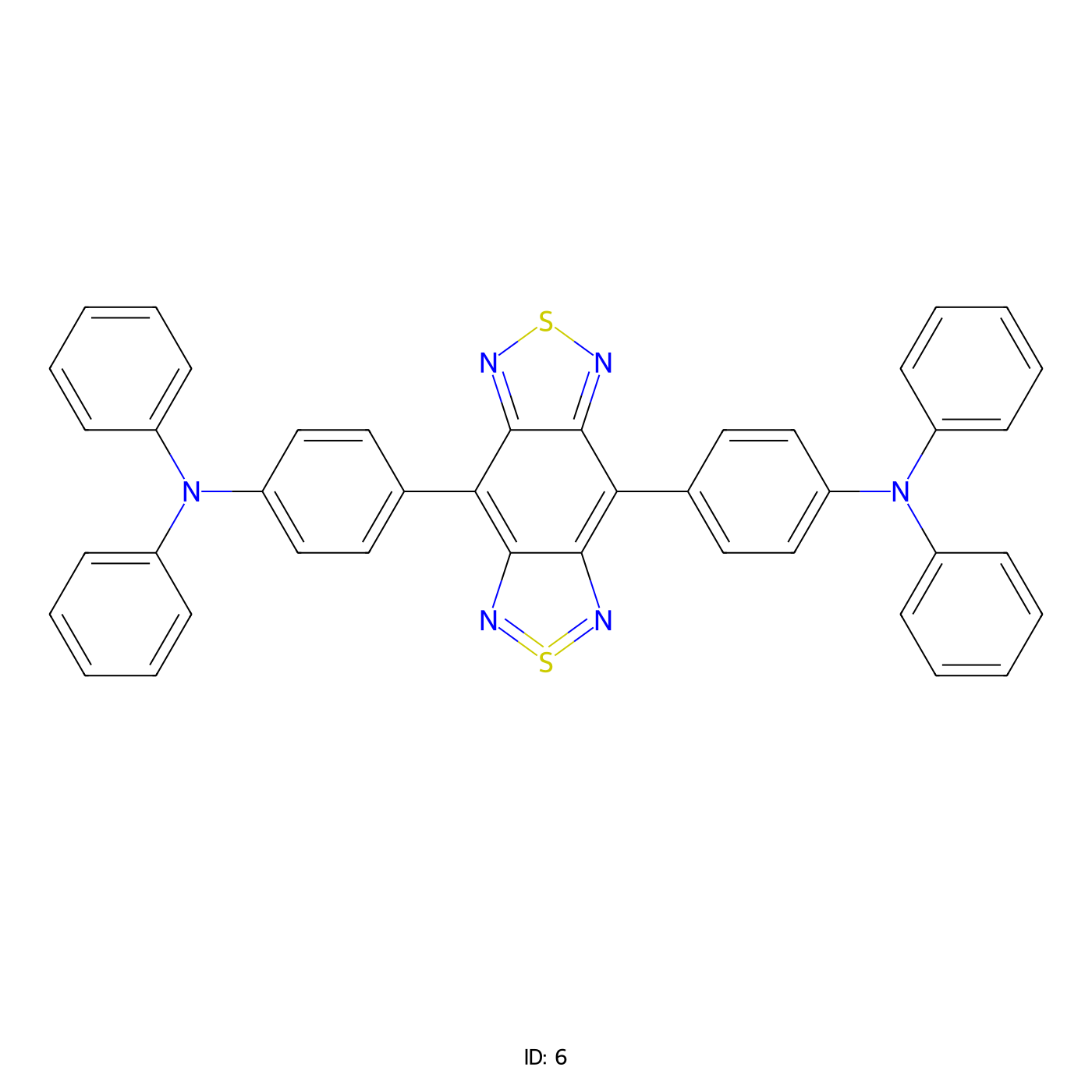 | 1 d 1 h 57 m 27 s | 1 m 5 s | 1431 |
| 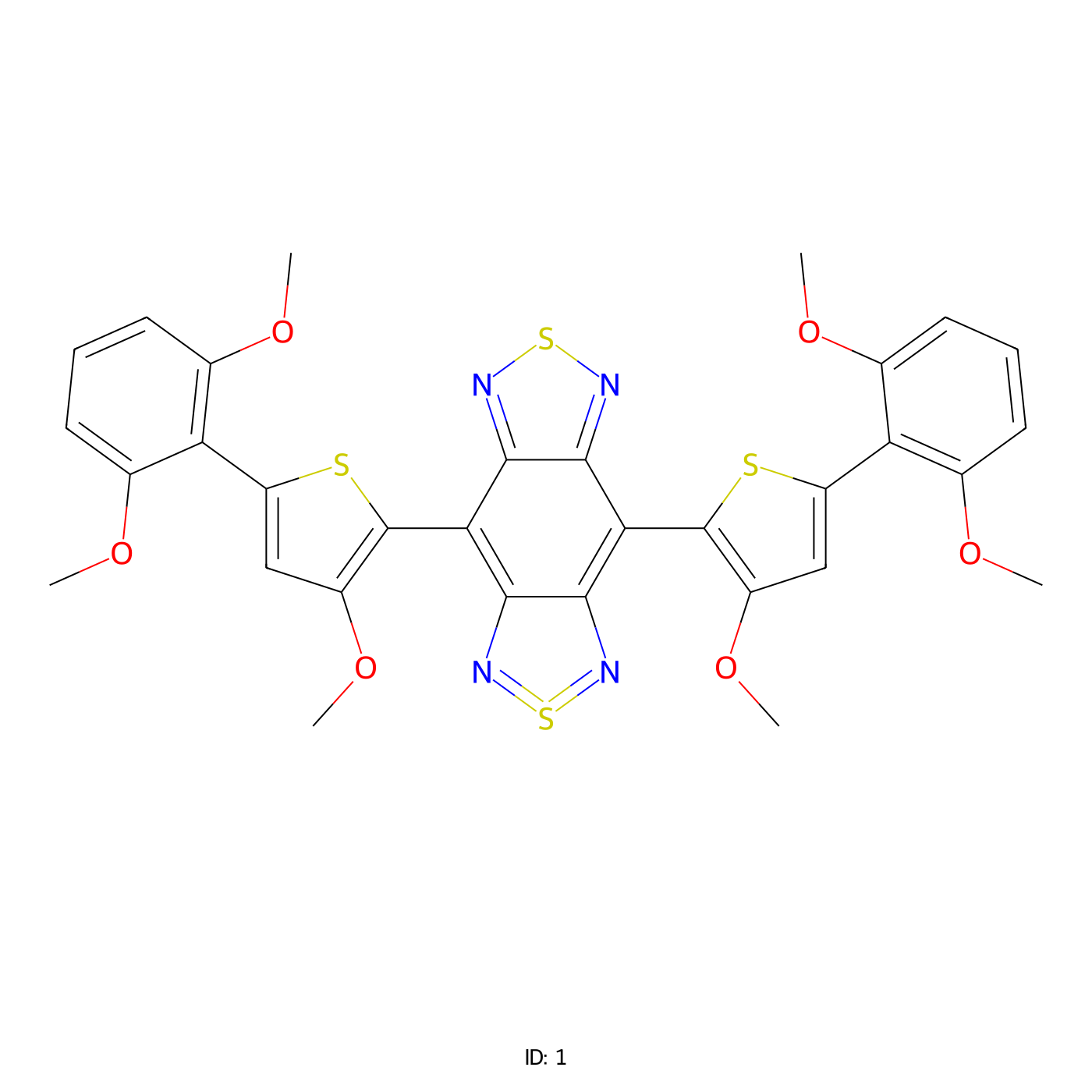 | 4 d 6 h 34 m 37 s | 1 m 5 s | 5681 |
| 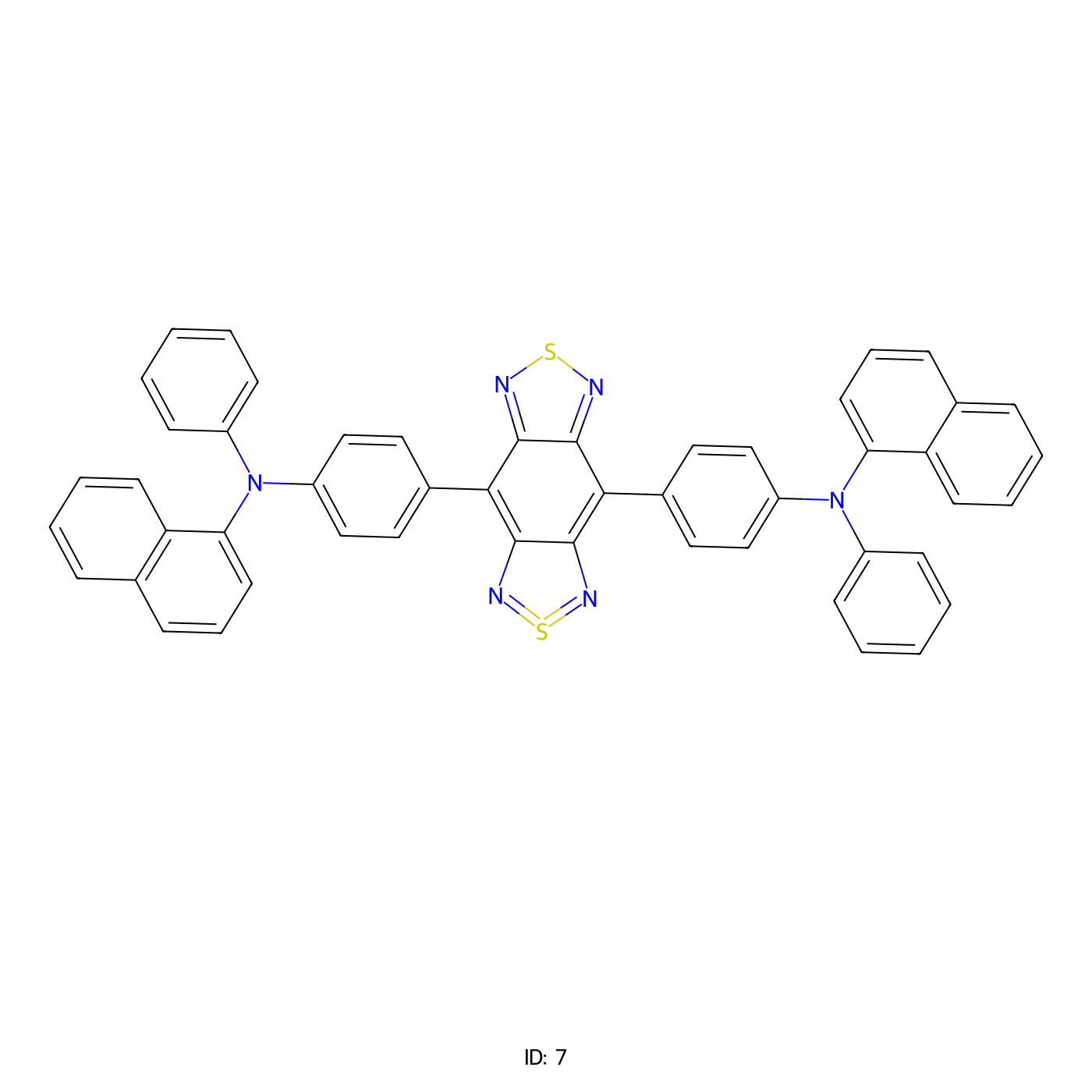 | 1 d 16 h 39 m 31 s | 1 m 5 s | 2252 |
| 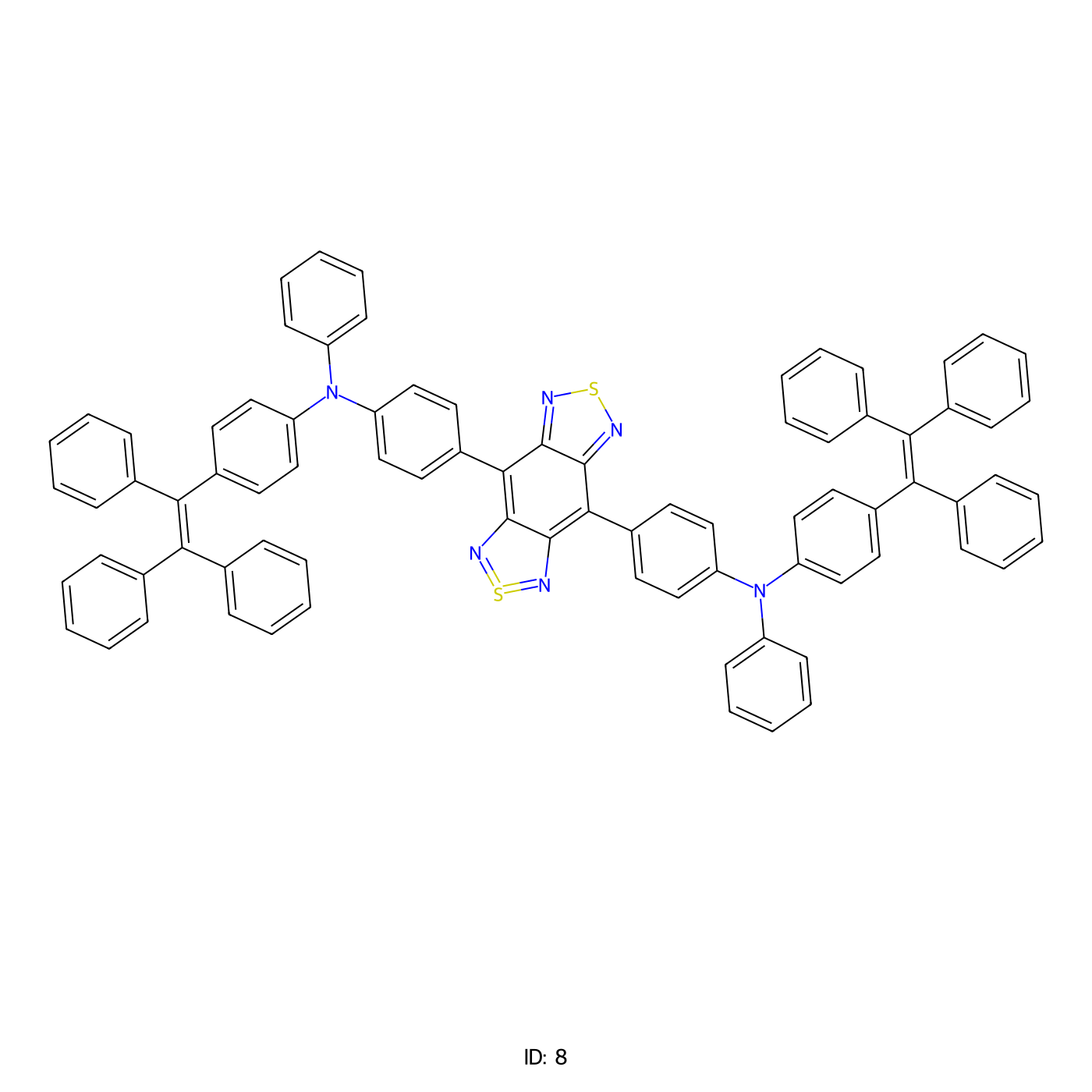 | 8 d 19 h 34 m 13 s | 44 s | 17310 |
| 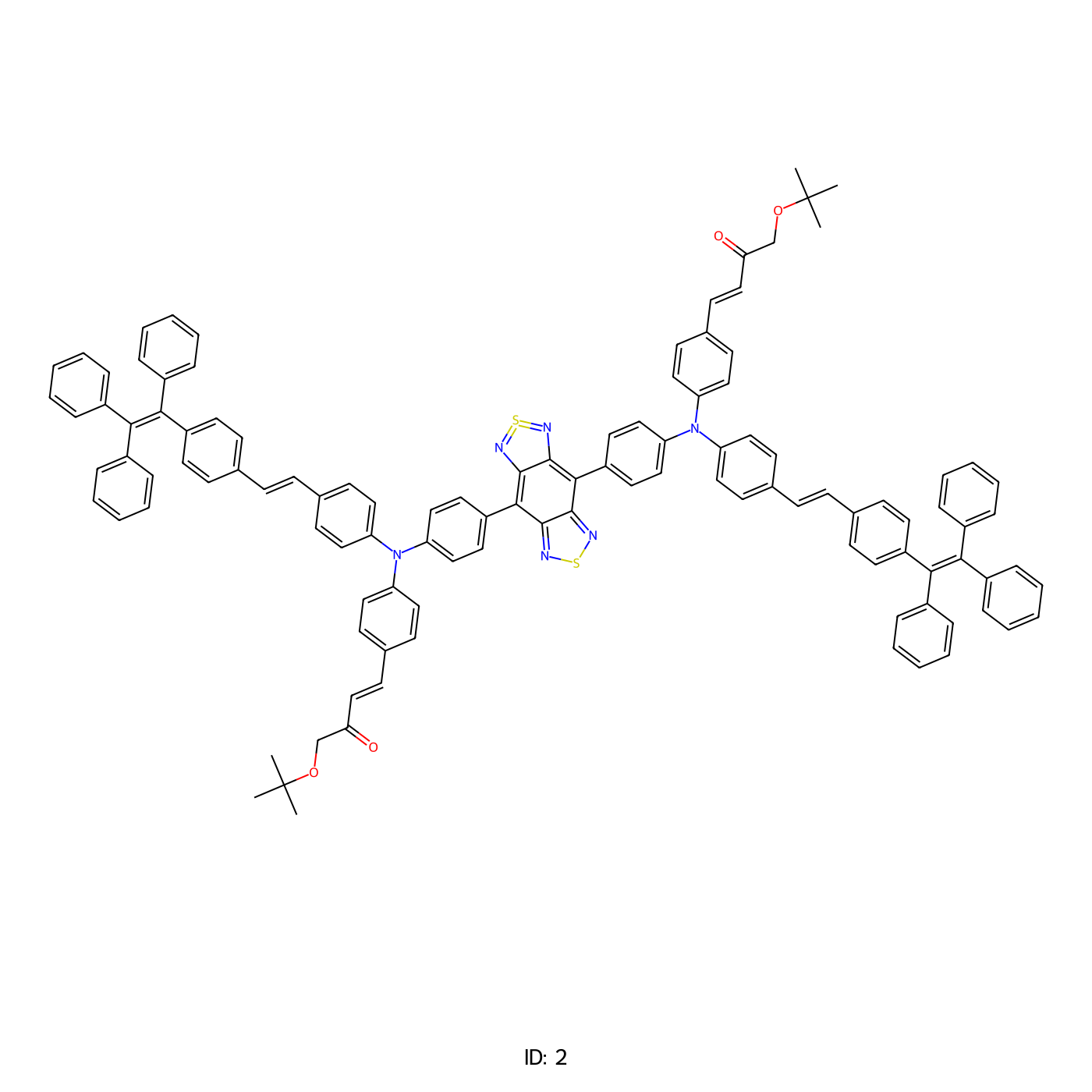 | 14 d 7 h 54 m 51s | 1 m 55 s | 10766 |
| 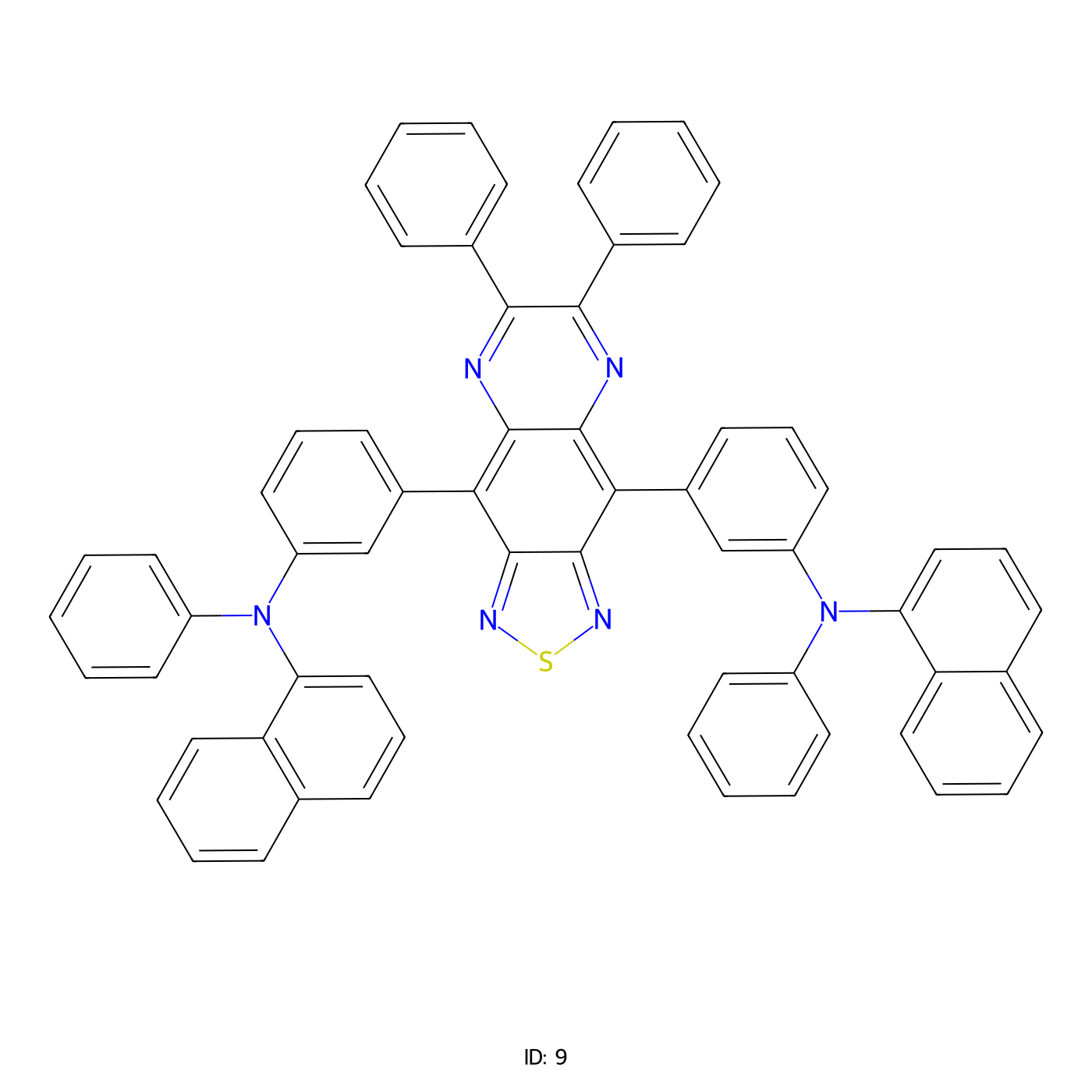 | 5 d 18 h 45 m 8 s | 1 m 29 s | 5612 |
| 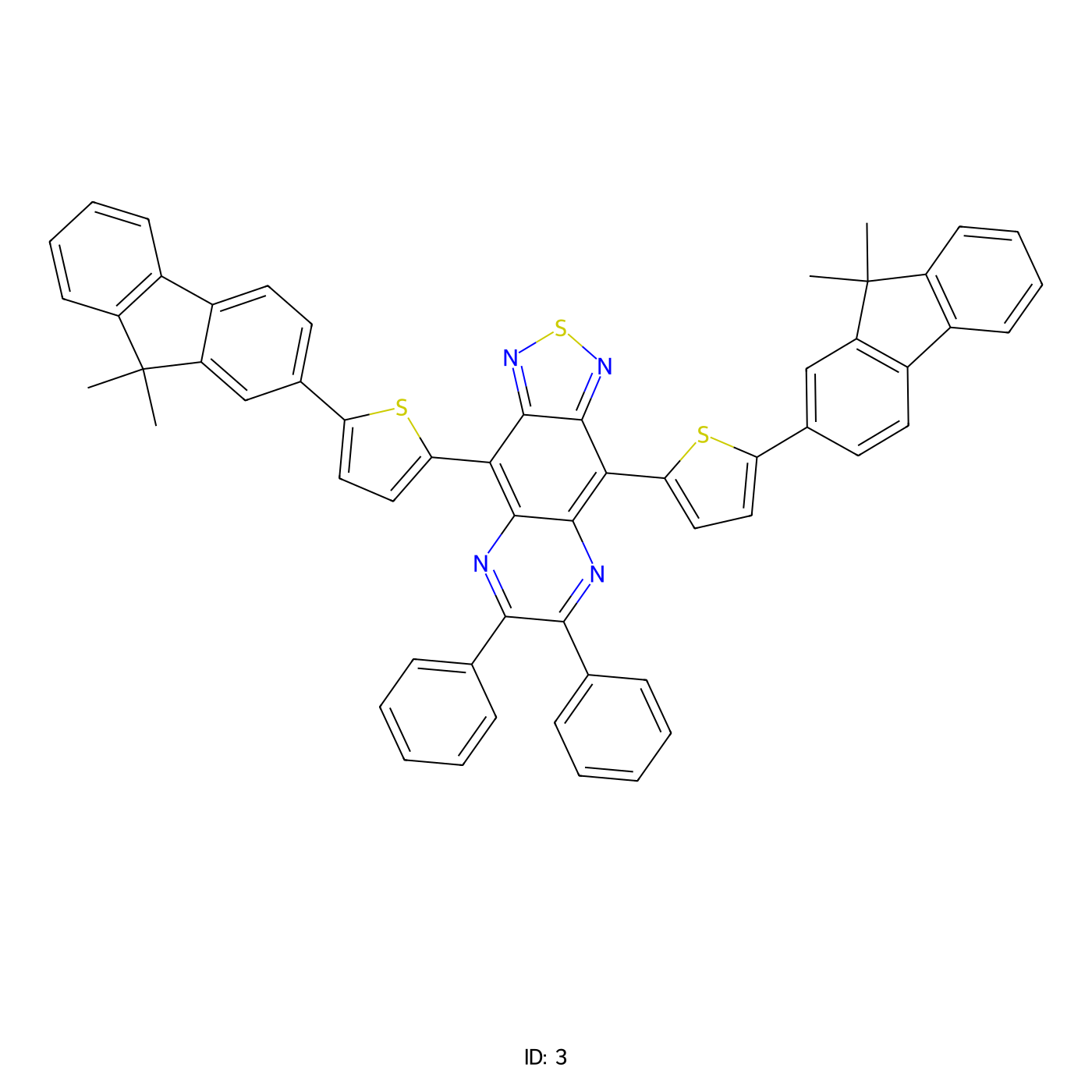 | 3 d 21 h 52 m 10 s | 34 s | 9939 |
| 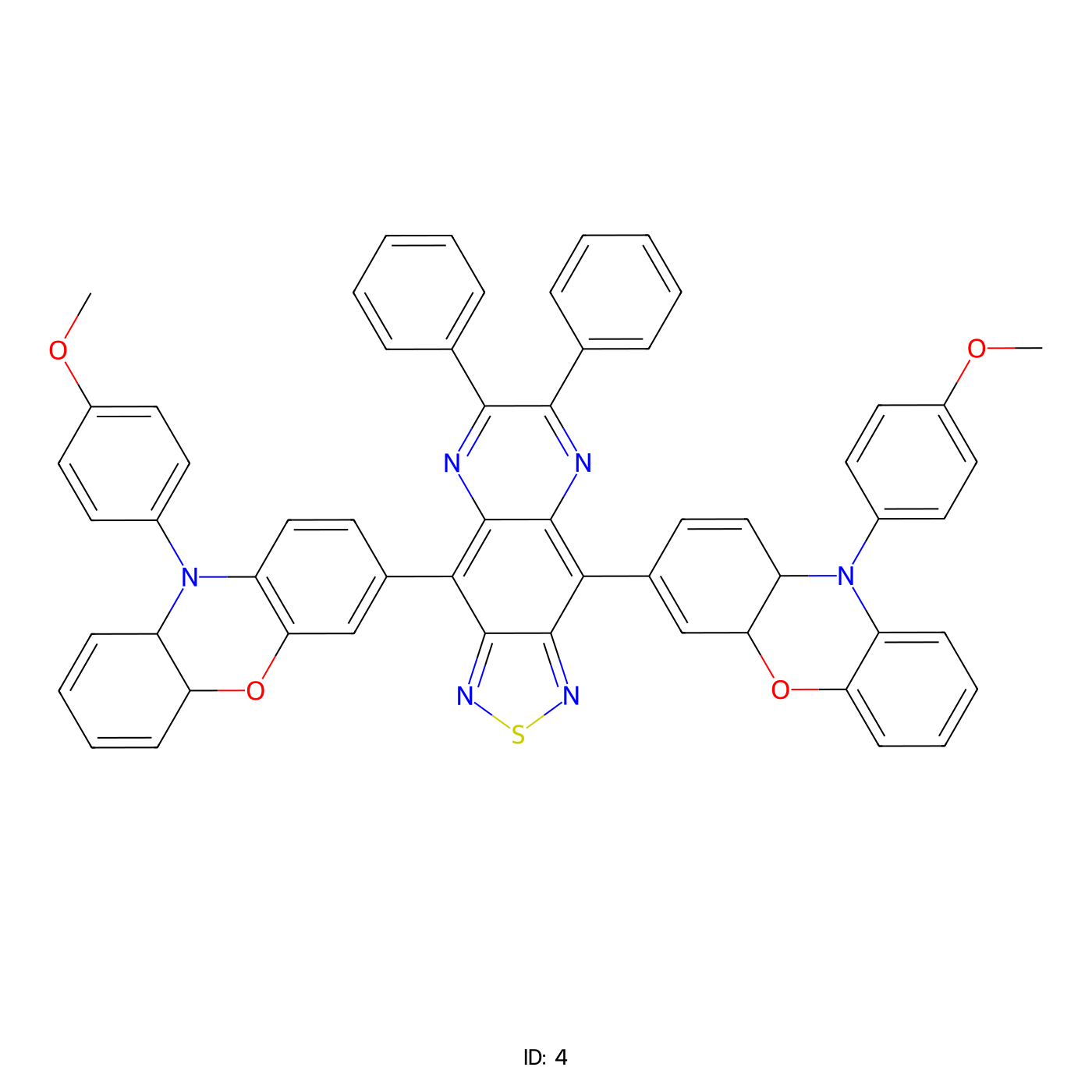 | 5 d 10 h 51 m 2 s | 1 m 1 s | 7722 |
| 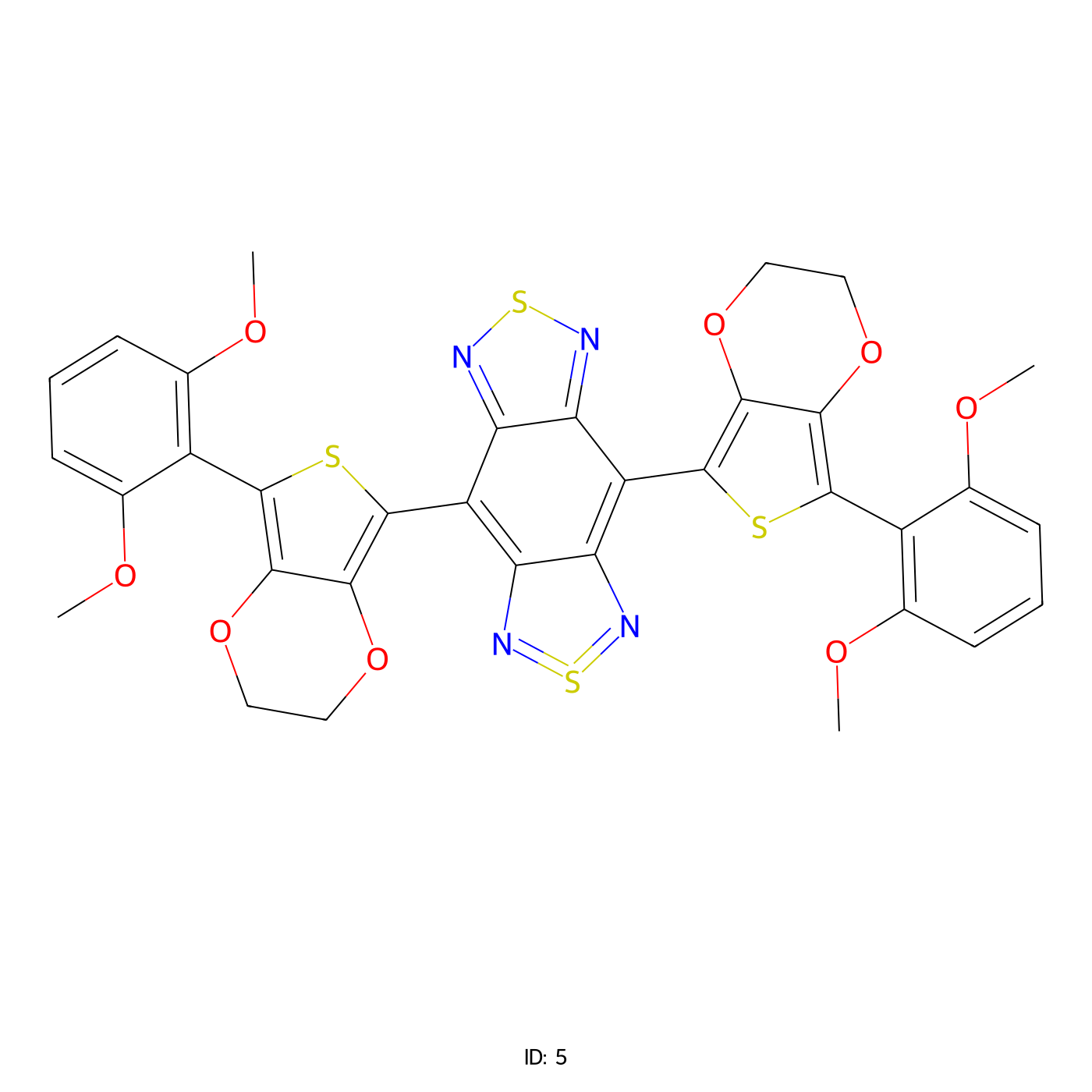 | 9 d 2 h 14 m 22 s | 1 m | 9939 |
| 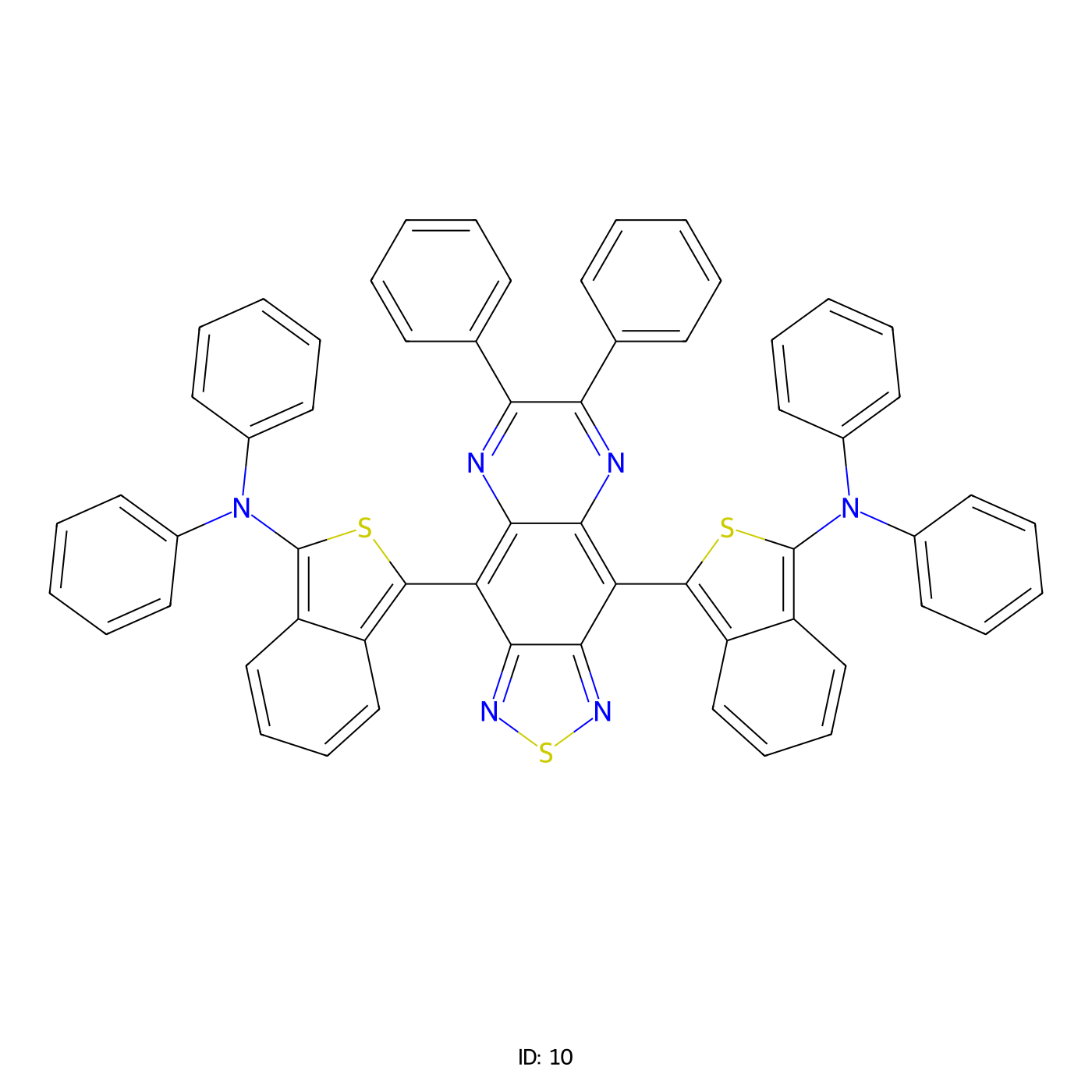 | 5 d 3 h 29 m 9 s | 33 s | 7722 |


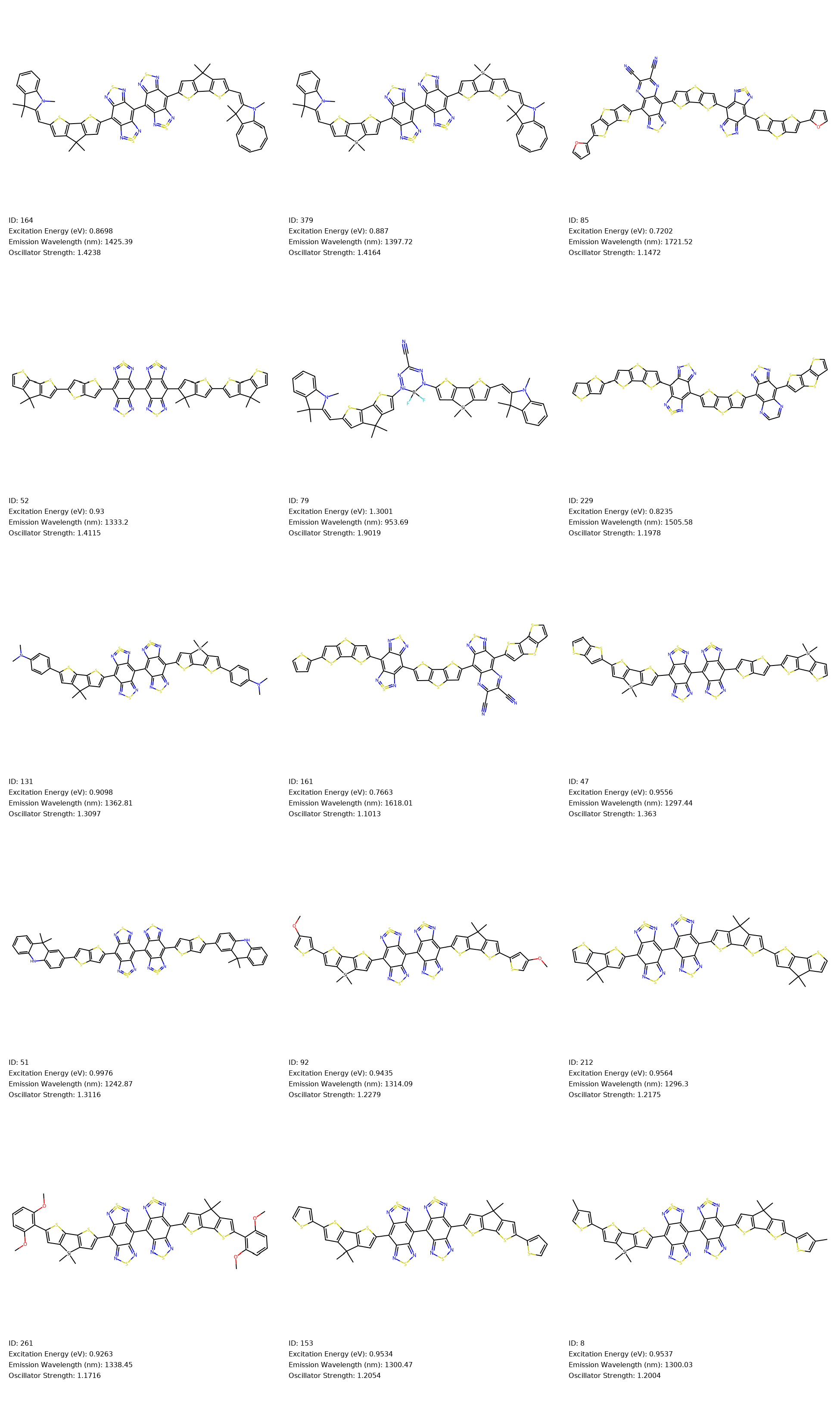


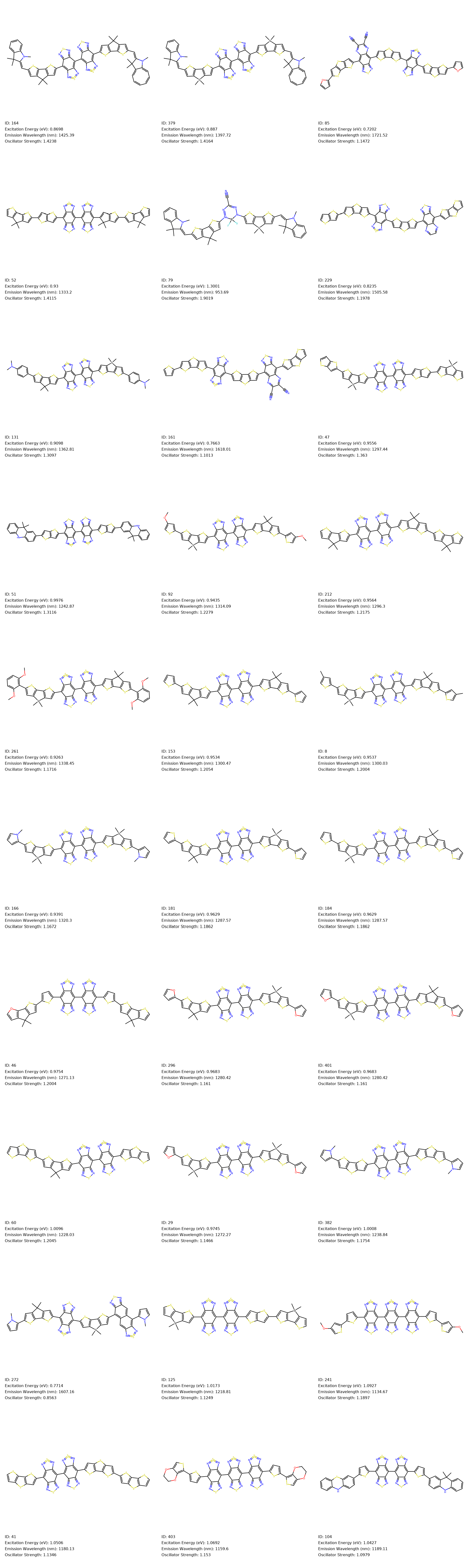


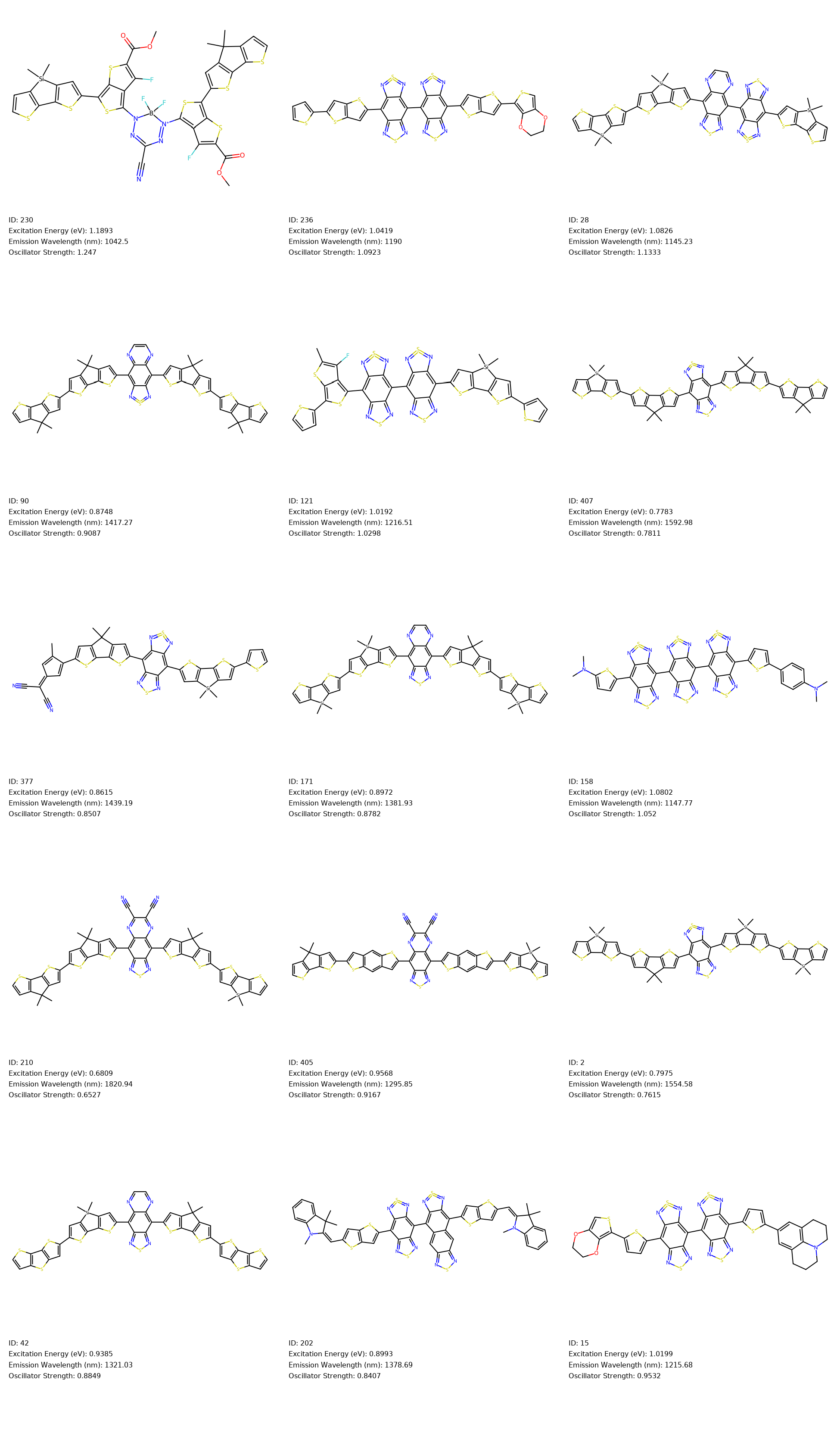


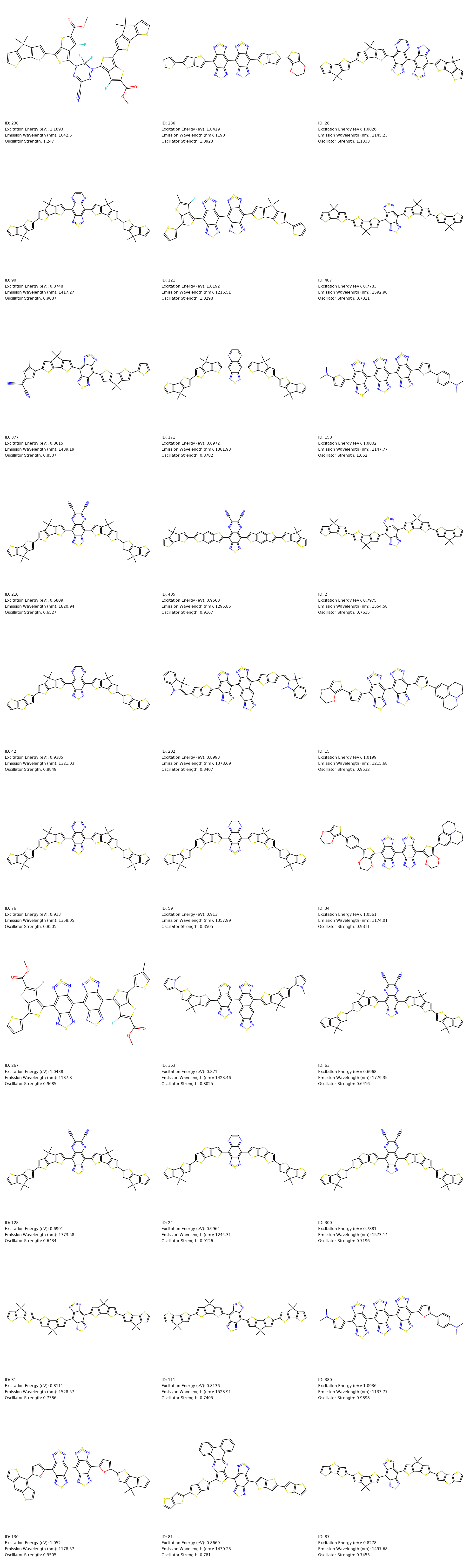


Figure S5. The top 60 generated molecules ranked by the product of emission wavelength (λ) and oscillator strength (ƒ).

Table S3. Abbreviation and IUPAC Names for the listed molecules in Figure 5.

| Entry | Abbreviation | Full Chemical Name (Components Separated by "/") |
| --- | --- | --- |
| 90 | DTP-DTP-TQ-DTP-DTP | 4,4-dimethyl-4H-cyclopenta[2,1-b:3,4-b']dithiophene / 4,4-dimethyl-4H-cyclopenta[2,1-b:3,4-b']dithiophene / [1,2,5]thiadiazolo[3,4-g]quinoxaline / 4,4-dimethyl-4H-cyclopenta[2,1-b:3,4-b']dithiophene / 4,4-dimethyl-4H-cyclopenta[2,1-b:3,4-b']dithiophene |
| 15 | EDOT-Th-BBT-BBT-Th-Jul | 2,3-dihydrothieno[3,4-b][1,4]dioxine / thiophene / 1H,5H-benzo[1,2-c:4,5-c']bis([1,2,5]thiadiazole) / 1H,5H-benzo[1,2-c:4,5-c']bis([1,2,5]thiadiazole) / thiophene / 2,3,6,7-tetrahydro-1H,5H-pyrido[3,2,1-ij]quinoline |
| 79 | TMI-DTS-DFB-DTP-TMI | 1,2,3,3-tetramethylindoline / 4,4-dimethyl-4H-silolo[3,2-b:4,5-b']dithiophene / 3,3-difluoro-2,3-dihydro-1H,5H-[1,2,4,5,3λ⁴]tetrazaborinine-6-carbonitrile / 4,4-dimethyl-4H-cyclopenta[2,1-b:3,4-b']dithiophene / 1,2,3,3-tetramethylindoline |
| 285 | MCz-DTP-TBO-DTP-MCz | 9-methyl-9H-carbazole / 4,4-dimethyl-4H-cyclopenta[2,1-b:3,4-b']dithiophene / 1H,5H-[1,2,5]thiadiazolo[3',4':4,5]benzo[1,2-c][1,2,5]oxadiazole / 4,4-dimethyl-4H-cyclopenta[2,1-b:3,4-b']dithiophene / 9-methyl-9H-carbazole |
| 164 | TMCP-DTP-BBT-BBT-DTP-TMI | (3aZ,9E)-1,2,3,3-tetramethyl-2,3-dihydro-1H-cycloocta[b]pyrrole / 4,4-dimethyl-4H-cyclopenta[2,1-b:3,4-b']dithiophene / 1H,5H-benzo[1,2-c:4,5-c']bis([1,2,5]thiadiazole) / 1H,5H-benzo[1,2-c:4,5-c']bis([1,2,5]thiadiazole) / 4,4-dimethyl-4H-cyclopenta[2,1-b:3,4-b']dithiophene / 1,2,3,3-tetramethylindoline |
| 377 | MCPM-DTP-BBT-DTS-Th | 2-(3-methylcyclopenta-2,4-dien-1-ylidene)malononitrile / 4,4-dimethyl-4H-cyclopenta[2,1-b:3,4-b']dithiophene / 1H,5H-benzo[1,2-c:4,5-c']bis([1,2,5]thiadiazole) / 4,4-dimethyl-4H-silolo[3,2-b:4,5-b']dithiophene / thiophene |
| 161 | Th-DTT-BBT-DTT-TQDC-DTT | thiophene / dithieno[3,2-b:2',3'-d]thiophene / 1H,5H-benzo[1,2-c:4,5-c']bis([1,2,5]thiadiazole) / dithieno[3,2-b:2',3'-d]thiophene / [1,2,5]thiadiazolo[3,4-g]quinoxaline-6,7-dicarbonitrile / dithieno[3,2-b:2',3'-d]thiophene |
| 81 | TT-TT-DBTQ-BBT-TT-TT | thieno[3,2-b]thiophene / thieno[3,2-b]thiophene / dibenzo[f,h]thieno[3,4-b]quinoxaline / 1H,5H-benzo[1,2-c:4,5-c']bis([1,2,5]thiadiazole) / thieno[3,2-b]thiophene / thieno[3,2-b]thiophene |
| 241 | MeOTh-Th-BBT-BBT-BBT-Th-MeOTh | 3-methoxythiophene / thiophene / 1H,5H-benzo[1,2-c:4,5-c']bis([1,2,5]thiadiazole) / 1H,5H-benzo[1,2-c:4,5-c']bis([1,2,5]thiadiazole) / 1H,5H-benzo[1,2-c:4,5-c']bis([1,2,5]thiadiazole) / thiophene / 3-methoxythiophene |

Footnote:

The abbreviations are constructed by connecting the acronyms of the individual building blocks with hyphens, indicating the sequence from the donor to the acceptor moieties. A list of the component acronyms is provided below:

- MCz: 9-methyl-9H-carbazole
- DTP: 4,4-dimethyl-4H-cyclopenta[2,1-b:3,4-b']dithiophene
- TBO: 1H,5H-[1,2,5]thiadiazolo[3',4':4,5]benzo[1,2-c][1,2,5]oxadiazole
- BBT: 1H,5H-benzo[1,2-c:4,5-c']bis([1,2,5]thiadiazole)
- TMI: 1,2,3,3-tetramethylindoline
- TMCP: (3aZ,9E)-1,2,3,3-tetramethyl-2,3-dihydro-1H-cycloocta[b]pyrrole
- MCPM: 2-(3-methylcyclopenta-2,4-dien-1-ylidene)malononitrile
- DTS: 4,4-dimethyl-4H-silolo[3,2-b:4,5-b']dithiophene
- Th: thiophene
- DTT: dithieno[3,2-b:2',3'-d]thiophene
- TQDC: [1,2,5]thiadiazolo[3,4-g]quinoxaline-6,7-dicarbonitrile
- TT: thieno[3,2-b]thiophene
- DBTQ: dibenzo[f,h]thieno[3,4-b]quinoxaline
- MeOTh: 3-methoxythiophene
- EDOT: 2,3-dihydrothieno[3,4-b][1,4]dioxine
- Jul: 2,3,6,7-tetrahydro-1H,5H-pyrido[3,2,1-ij]quinoline
- DFB: 3,3-difluoro-2,3-dihydro-1H,5H-[1,2,4,5,3λ⁴]tetrazaborinine-6-carbonitrile
- TQ: [1,2,5]thiadiazolo[3,4-g]quinoxaline


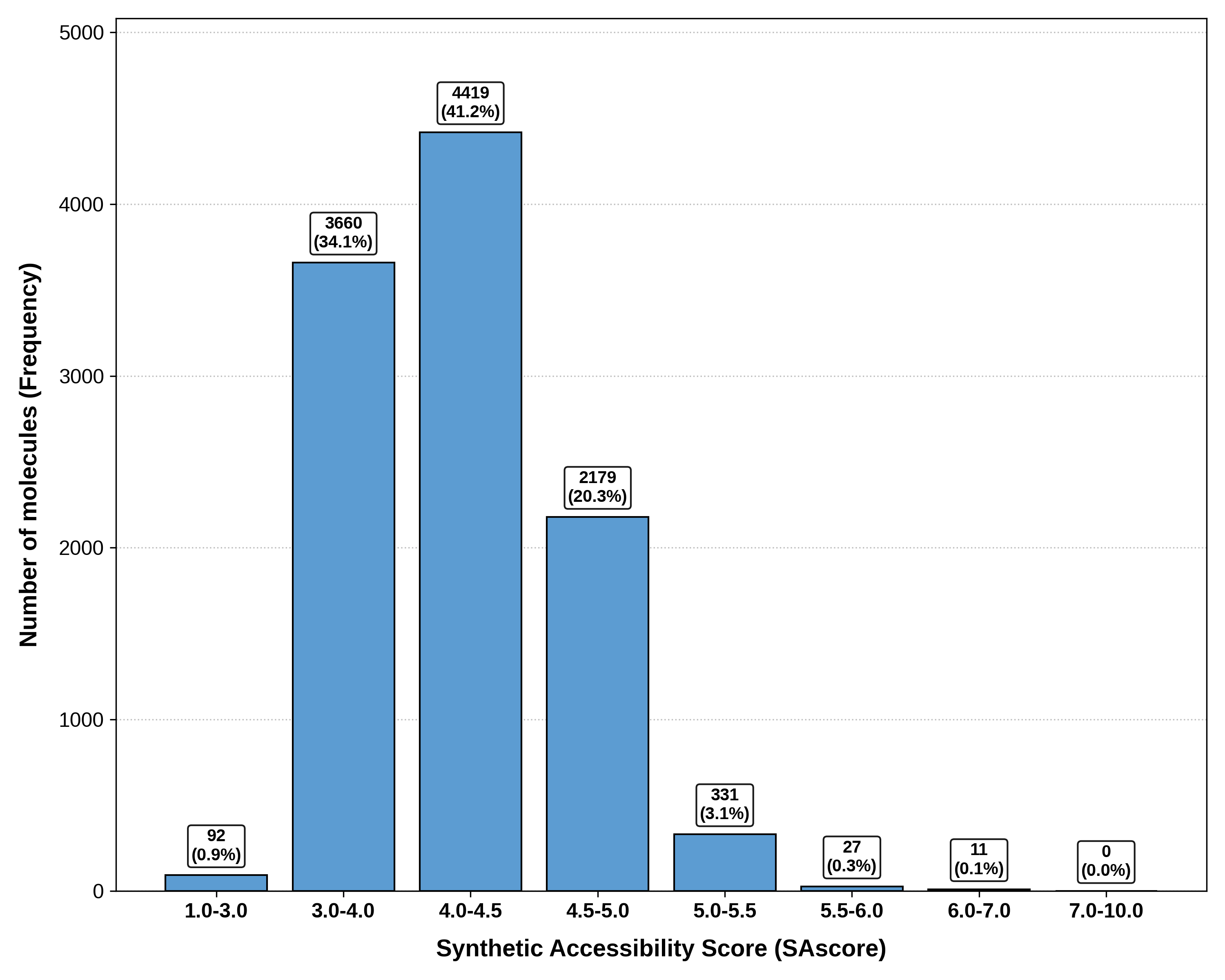


Figure S6. Distribution of the Synthetic Accessibility Score (SAScore) for the AI-generated NIR-II molecular pool consisting of 10,719 molecules evaluated. The SAScore provides a heuristic estimation of the ease of synthesis computed via RDKit, ranging from 1 to 10, where lower scores indicate higher synthetic feasibility. The prominent peak within the 3.0-4.5 range and the fact that over 96.5% of the structures score below 5.0 comprehensively confirm the high practical retrosynthetic viability of the generated candidates, effectively demonstrating the advantages of the fragment-based generative strategy.
