# Supplementary material for: Smart molecular design of NIR‐II organic fluorophores through self‐driven iterative evolution, deep learning, and fragment‐based assembly
Source: Smart Mol. 2026 Jun 10:e70064. Online ahead of print. doi: 10.1002/smo2.70064 (PMC13398758; doi:10.1002/smo2.70064)
Supplement: Supplementary file 2 — Supporting Information S2 [file SMO2-9999-0-s001.docx]

Supplementary video demonstration of accessible online interface for AI4NIR-II 1.0.
